# A Multitasking-Oriented Robot Arm Motion Planning Scheme Based on Deep Reinforcement Learning and Twin Synchro-Control

**DOI:** 10.3390/s20123515

**Published:** 2020-06-21

**Authors:** Chuzhao Liu, Junyao Gao, Yuanzhen Bi, Xuanyang Shi, Dingkui Tian

**Affiliations:** 1 Intelligent Robotics Institute, School of Mechatronical Engineering, Beijing Institute of Technology, 5 Nandajie, Zhongguancun, Haidian, Beijing 100081, China; 3120150091@bit.edu.cn (C.L.); 3120190150@bit.edu.cn (Y.B.); 3120170111@bit.edu.cn (X.S.); 3120170099@bit.edu.cn (D.T.); 2 Beijing Advanced Innovation Center for Intelligent Robots and Systems, Beijing 100081, China

**Keywords:** deep reinforcement learning, twin synchro-control, humanoid robot

## Abstract

Humanoid robots are equipped with humanoid arms to make them more acceptable to the general public. Humanoid robots are a great challenge in robotics. The concept of digital twin technology complies with the guiding ideology of not only Industry 4.0, but also Made in China 2025. This paper proposes a scheme that combines deep reinforcement learning (DRL) with digital twin technology for controlling humanoid robot arms. For rapid and stable motion planning for humanoid robots, multitasking-oriented training using the twin synchro-control (TSC) scheme with DRL is proposed. For switching between tasks, the robot arm training must be quick and diverse. In this work, an approach for obtaining a priori knowledge as input to DRL is developed and verified using simulations. Two simple examples are developed in a simulation environment. We developed a data acquisition system to generate angle data efficiently and automatically. These data are used to improve the reward function of the deep deterministic policy gradient (DDPG) and quickly train the robot for a task. The approach is applied to a model of the humanoid robot BHR-6, a humanoid robot with multiple-motion mode and a sophisticated mechanical structure. Using the policies trained in the simulations, the humanoid robot can perform tasks that are not possible to train with existing methods. The training is fast and allows the robot to perform multiple tasks. Our approach utilizes human joint angle data collected by the data acquisition system to solve the problem of a sparse reward in DRL for two simple tasks. A comparison with simulation results for controllers trained using the vanilla DDPG show that the designed controller developed using the DDPG with the TSC scheme have great advantages in terms of learning stability and convergence speed.

## 1. Introduction

Humanoid robots have recently become a focus in academic research. State-of-the-art humanoid robots are capable of working alongside humans. They can stabilize themselves in a practical work environment and use their arms for simple tasks, such as lifting a box, using power tools, and maintaining their balance. However, these tasks usually require a researcher or engineer to carefully design the trajectory for the robot. The quick generation of stable motion for various robot tasks is an important goal in both research and industry. Traditional robotic arm motion planning schemes are time-consuming and require researchers to have expertise in mathematics, kinematics, inverse kinematics, and other areas. As an alternative, deep reinforcement learning (DRL) can be applied to plan joint trajectories for robotic arms, mobile robots, and even quadruped robots. However, universal dual-arm robots usually take a long time to learn a specific action. A task-oriented arm trajectory design can take a few days or longer; multiple tasks require even more time. This time demand negatively affects user experience and hinders the adoption of humanoid robots. For safety and reliability, robot arm trajectory planning should be carried out for multiple tasks instead of messing around with the robot.

It is well known that DRL is extremely sensitive to hyperparameters and network structures. The performance of some DRL algorithms is worse than random behavior. Inaccurate hyperparameters result in unstable learning results or lack of convergence. With an imperfect network structure or inappropriate hyperparameters, the training process for robot arms is often unpredictable. For the analysis of experiments on hyperparameter sensitivity, see Section 5 below.

This problem can be solved in several ways. For example, the number of training samples or sampling complexity can be increased to reduce the deviation and variance of the neural network prediction output, or reward engineering can be used to bring the reward function output closer to the desired value. High sampling complexity often leads to a geometric increase in the number of calculations, which is undesirable for robot control. Reward engineering requires researchers to analyze the controlled object and target to obtain various characteristics, such as the Euclidean distance from the robot arm to a target point, which could affect the convergence of the network. It can also be difficult to apply traditional reward engineering for quickly planning motion trajectories for multiple tasks.

In the present study, we propose a scheme for rapid and accurate motion planning based on DRL with twin synchro-control (TSC). The effectiveness and reliability of the algorithm are verified using two simulations. In one simulation, a planar three-degree-of-freedom (3-DOF) manipulator is used for trajectory planning experiments on the end points of the arms. In the other simulation, humanoid robot arms are controlled in a three-dimensional (3D) simulation environment to plan arm joint motion and rotate a vehicle steering wheel by 30°. We also use a model of the humanoid robot BHR to verify the feasibility of our algorithm in a simulation.

**Figure 1:**
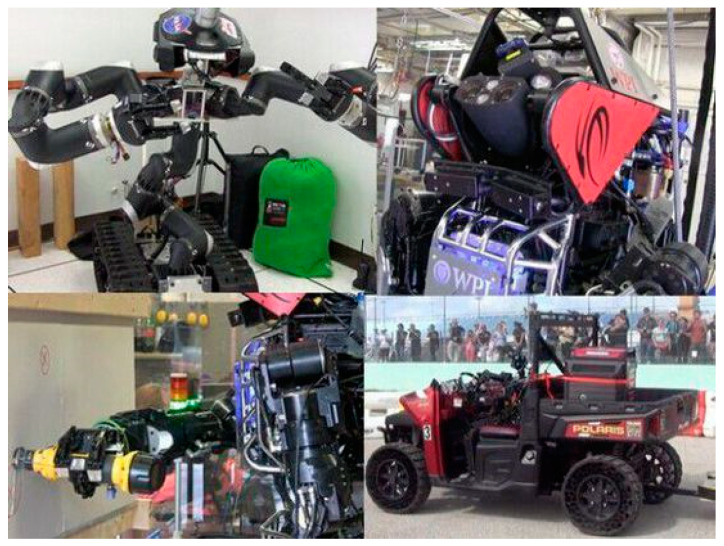
Photographs from the DARPA Robotics Challenge.

Robots that can work in a human environment have been developed to help people perform various tasks, such as search and rescue in a coal mine [1] and disaster ground information collection [2]. Atlas (Boston Dynamics, Waltham, MA, USA) is an advanced humanoid robot whose control system coordinates the motions of the arms, torso, and legs to achieve whole-body mobile manipulation, greatly expanding its reach and workspace. Atlas can balance itself while performing tasks, allowing it to work in a large volume while occupying only a small footprint. Atlas participated in the Defense Advanced Research Projects Agency (DARPA, Arlington County, VA, USA) Robotics Challenge (DRC) sponsored by the US Department of Defense. The purpose of the competition was to develop robots that can perform a first-aid mission in a populated environment with hazards, such as the Fukushima Daiichi Nuclear Power Plant. The event included climbing ladders, opening doors, removing rubbish in front of a door, walking on rugged roads, breaking walls, connecting to fire hydrants, closing leaking valves, and driving cars. Figure 1 shows photographs of robots that participated in the DRC.

Designing control algorithms for these robots is extremely challenging. From a control engineering perspective, these humanoid robots are high-dimensional non-smooth systems with many physical constraints that make them move like a human. Each joint has a fixed range of motion. Analytical models of robots are often inaccurate and cause uncertainties in the dynamics. Sensor arrays and multi-layer software can introduce noise and delay in transmission. Conventional control theories are insufficient for various tasks. Robot joint trajectory planning is an extremely complex process restricted to experts. Some advanced control algorithms have been developed to deal with complex situations. They require parameter tuning and a lengthy design process.

A traditional humanoid robot control scheme depends on the accuracy of the system dynamics model used. Robots have uncertainties in joint friction, contact with the ground, and many other aspects, making it extremely difficult to accurately model in a real environment as its nonlinear and non-smooth dissipation. Most kinematics- and model-based robotic arms are thus unsuitable for rapid and accurate planning of joint trajectories for complex tasks.

To increase the versatility of a robot planning scheme and reduce the dependence on the system dynamics model, DRL is applied to the robot control system. Humans have experienced some periods for controlling their arms, including legs and other joint. Such as unconscious or conscious but not fully controlled swinging of the arm during infancy, and the period of arm with consciously controlled but not perfect or completing an action without enough knowledge. In the end, the arm can learn a lot and be able to implement a sufficiently robust arm control. Even if the required task changes, the training process can be carried out rapidly.

The gradient of a stochastic strategy is related to the expectations of states and actions. A large number of samples must be collected in the state space and the action space for the mean to approximate the expectation. In comparison, for a deterministic strategy, the actions are definitely and fewer samples are required, especially for agents with a large action space, such as humanoid robots. Because of the extremely large dimension of the action space in the present study, stochastic strategies would require a lot of samples in the action space; we thus adopt the deep deterministic policy gradient (DDPG) algorithm.

We designed and built the data acquisition system and proposed a TSC scheme with DRL for the motion control planning of humanoid robots. Joint trajectories are rapidity and stably obtained for a given application task. This approach uses a priori knowledge from a data acquisition system to accelerate the training of DRL agent. We compare the simulation results of a planar 3-DOF manipulator and a model of the BHR robot obtained using the DDPG algorithm with and without the TSC scheme.

A workflow diagram of the TSC scheme based on the data acquisition system is shown in Section 3.2. The data acquisition system captures the rotation angle of all human joints corresponding to the positions of the data acquisition shelf in real time. These recorded angle data are imported into the DDPG algorithm as a priori knowledge to speed up the training process. The learning-from-expert strategy methods are limited by the accuracy of samples. Poor performance was obtained on a test with DRL because some action-state pairs that did not appear when training the neural network were used. Therefore, we used reinforcement learning instead of imitate learning. Our scheme with a priori knowledge is suitable for most humanoid robots developed for application tasks. It does not need to match the sensor wearer’s body size and the data acquisition shelf. The data acquisition system can also be used with humanoid robots of various sizes to obtain a priori knowledge.

The rest of this paper is organized as follows: Section 2 reviews related work and highlights the contributions of this paper. Section 3 introduces the model of the BHR humanoid robot in the simulation environment, and then presents the TSC scheme, which consists of the data acquisition system platform and the DRL algorithm. Section 4 describes two simulation environments and applies the proposed methods to the simulation. Section 5 analyzes the experimental results of the two simulation environments. Section 6 summarizes the advantages and disadvantages of the proposed scheme and gives suggestions for future work. The last section lists the references cited in this article.

## 2. Related Work

### 2.1. Humanoid Robot Control Algorithms Based on Reinforcement Learning

Reinforcement learning algorithms can be classified into value-based, policy-based, and hybrid (e.g., actor-critic) algorithms. They attempt to find the optimal policy that maximizes the expected rewards. Kober et al. [3] reviewed robot reinforcement learning and suggested areas for future research. Q-learning [4] and Sarsa [5], whose policies and value function are represented by a two-dimensional (2D) lookup table indexed by state-action pairs [6], are typical value-based reinforcement learning algorithms. Duan et al. [7] presented a benchmark suite of continuous control tasks, including cart-pole swing-up, and some other tasks with high state and action, such as 3D humanoid locomotion based on partial observations and hierarchical structure. They also reported some novel findings on reinforcement learning algorithms. Many control methods with DRL have been proposed for humanoid balancing [8,9,10].

Humanoid robots are high-dimensional non-smooth systems with many physical constrains. To deal with increasingly sophisticated application scenes, deep learning can be used for humanoid robots to automatically learn the abstract representation of large-scale input data. This representation can then be used as a basis for reinforcement learning with self-motivation and optimize problem-solving strategies. DeepMind (Google) combines deep learning with perceptual ability and reinforcement learning with decision-making ability to create DRL. Well-known DRL algorithms for solving problems in continuous state and action spaces include Trust Region Policy Optimization (TRPO) [11], Normalized Advantage Function (NAF) [12], Asynchronous Advantage Actor Critic (A3C) [13], and DDPG [14]. as one DRL learning with a model-free algorithm based on deterministic policy that can handle very complex and dynamic motor tasks in continuous state and action spaces. DDPG was proposed in [14] as a robust algorithm for solving more than 20 simulated classic robot problems. Schulman et al. [15] attempted to combine the advantage function with the trust region optimization procedure for both the policy and the value function for robot control tasks in 3D scenes.

An application-oriented robotic arm that uses DRL with a general control policy has been proposed. Levine et al. [16] utilized a deep convolutional neural network to represent the strategy approximately, and used a guided policy search (GPS) scheme to guide a robot through some simple operations. The GPS method divides the strategy search method into two optimizations, namely trajectory optimization and policy optimization. The role of trajectory optimization is to generate high-quality data with traditional controllers or some stochastic optimization methods. Policy optimization uses supervised learning with the data generated from trajectory optimization [17]. Lavine et al. [18] also used deep neural network models to predict robot movements and achieved good results in robotic grabbing tasks based on hand-eye coordination. Abbeel et al. [19] proposed an apprenticeship learning method, in which an expert attempts to maximize a reward function that is expressed as a linear combination of known features, and proposed an algorithm for learning a task demonstrated by an expert to give robots the ability to learn tasks for which they were not programmed [20]. Some researchers have combined DRL with cameras as the input of deep neural networks to develop robotic arm control. Zhou et al. [21] used a boosting sample deep Q network method with a binocular stereoscopic vision system for humanoid action imitation research. Gu et al. [22] demonstrated that the off-policy training of deep Q-functions can be applied to 3D manipulation tasks (e.g., opening a door) for robots without prior demonstration.

### 2.2. Combining RL with Demonstrations

Several recent studies have noted that learning from demonstration (LfD) [23], a mechanism to learn from the experience of experts, is a promising means of overcoming the difficulties related to exploration in reinforcement learning (RL) [24]. DQfD [25] leverages sets of expert data to pre-train the weights of the DQN It uses temporal difference and supervised learning to classify the demonstrator’s actions to solve the problems of cold starts and the high cost of training. Unlike the DQfD, which is oriented toward discrete problems, DDPGfD [26] is applied to domains of continuous action. Similarly, the data for demonstration and those generated by agent are added to a replay buffer, and sampling ratio of the demonstrations to the transitions are automatically though a prioritized replay mechanism. POfD [27] is also a method of policy optimization that uses demonstrations to guide exploration through compel occupancy measure to match the learned policy with the given demonstrations. It trains the agent by using the TRPO in an environment of dense reward, and randomly samples imperfect trajectories to collect expert data. Neither the DQfD nor the DDPGfD can make full use of the demonstration-related data when the amount of expert data is insufficient and not highly correlated with the transition generated by the agent. The POfD introduces a more circuitous method of optimizing JS divergence to improve the LfD method in discrete action spaces. However, but the effectiveness of most such algorithms has been verified only in the gym environment. The DAPG [28] as a model-free DRL can be scaled up to multipletasks using a 24-DoF hand in simulations, and can significantly reduce sample complexity using only a few demonstrations by a human. It can achieve a higher success rate than the DDPGfD algorithm in each of the four hand tasks.

The method proposed here is completely different from DDPGfD, DQfD, and POfD. The author of the DDPGfD proposes a ranking-based sampling replay buffer between greedily prioritizing and uniformly randomly sampling to expert data and added L2 regularization to actor-critic (AC) network parameters to replace manual shaping reward. The DQfD is pre-trained on the demonstration data from a quasi-dedicated controller that updates the network based on four losses to solve the problem of cold start in Q-learning. The POfD is identical to the previous two articles and focuses on solving the problem of sparse reward signals during exploration. The developer introduced JS divergence to measure the distance between the teaching trajectory of the expert strategy and the trajectory trained by the agent and transforms the objective into a new target defined as a measure of occupancy. It also establishes a connection between the optimized lower bound and confrontation generation training, and alternately optimizes the two sub-processes. Research on the DAPG usingmanipulators with 24 degrees of freedom is similar to that on the DDPGfD. The author used action cloning to cause strategies to self-execute and integrated teaching into the reward function. This is also the basis for the method proposed here. The main purpose of our research is to quickly deploy acceptable or basically acceptable expert strategies (demo actions) through robots.

### 2.3. Neural Network Control Learning of Manipulator

Robotic arm control methods have attracted increasing research attention. Liu et al. [29] presented an adaptive neural control scheme that considers the unknown output delay and computational efficiency, and used the Lyapunov stability principle to prove the convergence of their method. For the real-time optimization of the trajectory of a manipulator, Jin et al. [30] established a dynamic neural network to recursively calculate the operability-maximum control action of the redundant manipulator under physical constraints. Li et al. [31] proposed a primitive dual neural network considering noise control. The designed neural controller can accurately track the reference trajectory in a noisy environment by using harmonic signals to eliminate unknown amplitude and phase information. In [32], Yang et al. developed a robot control/recognition scheme that improves the convergence velocity to identify unknown kinematics and dynamic parameters of the robot, and improved the performance of estimation by integrating the parameter estimation error information into the recognition algorithm. Brahmi et al. [33] proposed a solution based on human upper limb inverse kinematics and achieved robust nonlinear control for a 7-DOF manipulator. Yi et al. [34] proposed a self-driven joint model that combines articulated arm coordinate measuring machine joints with mechanical arms to achieve automatic rotation. Robotic arms are also used in powered exoskeleton and brain and cognitive neuroscience research. Leigh et al. [35] studied the ability of patients with long-standing tetraplegia to use interface-system-based robotic arm control to perform 3D reach and grasp movements. The results suggest that humans can recover a portion of the central nervous system after several years of damage to control a robot arm.

### 2.4. Digital Twin Technology with Synchro-Control

The “twins” concept in manufacturing dates back to the Apollo Program [36]. To rapidly, securely, and accurately train astronauts and perform simulation experiments, NASA built two identical space vehicles. The space vehicle that remained on earth, called the twin, was used to mirror the conditions of the space vehicle during the mission.

Digital twin technology has been applied in the field of robotics. Verner et al. [37] proposed an approach in which robotics projects focus on the development of learning robots to expedite the process; it is a reinforcement learning method where a virtual twin of a humanoid robot learns to lift a weight of unknown mass through an autonomous trial-and-error search. Laaki et al. [38] developed a digital twin prototype to analyze the requirements of communication in a mission-critical application, and verified its low latency and high levels of security and reliability via a system comprised of a robotic arm and a virtual reality system with a 4G mobile network. Spranger et al. [39] proposed a solution for the programming and training of remote robots without imposing delays and inefficiency with a vision-based 3D hand detection and gesture recognition subsystem; a digital twin of the robot is simulated as visual feedback.

The term synchro-control (synchronous control) comes from the field of automatic control theory. It has been applied in other fields, such as medical science [40], computer science [41], and fieldbus technology [42]. It has recently been introduced into the field of robotics, for example, the control of wheeled robots [43,44] and pneumatic systems [45].

## 3. Preliminaries

### 3.1. Humanoid Robot

**Figure 2:**
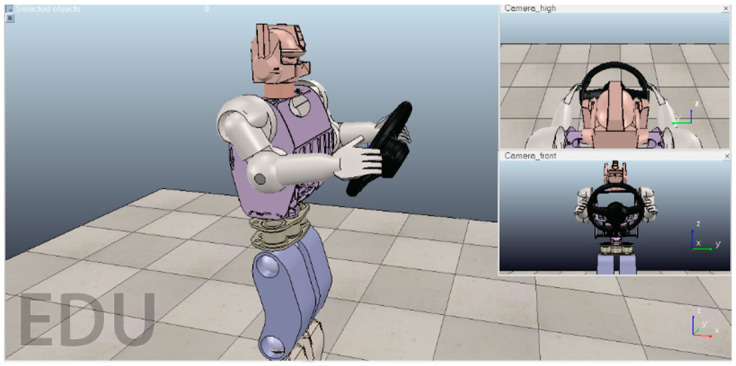
Screenshot of the robot simulation environment.

We used a simulation environment to build a robot model. The V-REP simulation environment is shown in Figure 2. The two small windows on the right are images captured by surveillance cameras at different angles. The steering wheel is located about 15 cm in front of the robot’s chest, and the angle with respect to 30° from the plane vertical. The upper arms of the robot are lifted up from perpendicular to the plane by 30°. The lower arms are then raised by 70°. The palms can naturally touch the steering wheel in this pose, as shown in Figure 2. We consider this pose of the arms to be the robot zero position for planning the arm joint trajectories.

**Figure 3:**
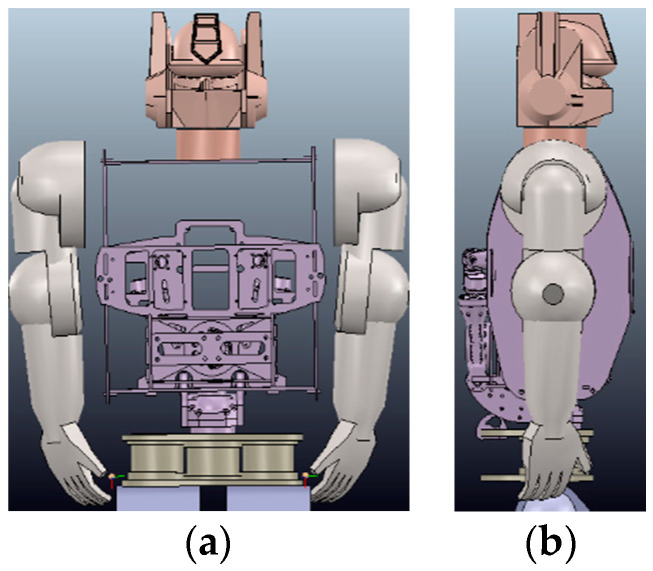
Simulation model of the BHR robot. (**a**) Front and (**b**) side views.

In the second simulation experiment, we studied a motion trajectory planning and control problem for the BHR humanoid robot. The simulation experiment was performed in a 3D environment. We used the remote application programming interface (API) framework of the robot dynamics simulator V-REP to achieve real-time simulation, where V-REP was used as the server and a Python integrated development environment (IDE, Spyder) was used as the client. The synchronous mode of V-REP communication was used to meet the real-time requirements. Each arm in the BHR robot has three joints: two for the shoulders, for pitch and roll, and one for the elbow, for pitch. Between the two arms is a chest cavity composed of carbon fiber boards. The upper body structure of the robot is shown in Figure 3.

### 3.2. Twin Synchro-Control Scheme

Typical approaches for designing the reward function lead to sparse rewards. Even though sparse rewards can promote learning, they are usually not desirable because they can result in extremely slow training of DRL agents. The lack of positive sample rewards makes learning difficult to stabilize, making training convergence difficult. An alternative is to set the rewards more carefully, add outsmart reward conditions, or adjust existing reward factors until the robot no longer takes shortcuts to achieve the goal.

We propose a robot joint trajectory planning scheme for multitasking-oriented scenarios called the TSC scheme, which is a knowledge-based method. To mitigate the problem of sparse rewards, we designed a data acquisition system to record the arm joints of the demonstrator and incorporate demonstration into the DRL algorithm these data are used to make the learning algorithm faster and more accurate. Although the robot’s DRL algorithm is designed for any finite Markov decision process, we only need to solve real-world tasks. We thus use the TSC scheme to create a series of prior state-action pairs in the real world, so that humanoid robots can quickly learn multiple tasks in simulation environments via DRL. The angles of joints in the simulation are recorded on a computer and sent to the motor driver with a BHR control cycle of 4 ms to perform the position control of the robot joints. TSC is used to add more learning signals to solve the problem of insufficient information due to sparse learning. We use the DDPG algorithm as the learning algorithm because it performs very well for continuous action in high-dimensional space. The basic principles of TSC are described in Section 3.2.1 and Section 3.2.2.

**Figure 4:**
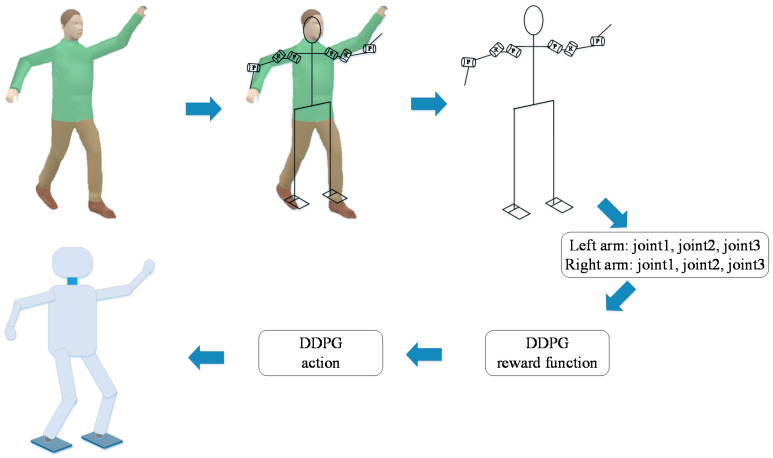
Workflow chart of the TSC scheme.

The workflow of the TSC scheme is shown in Figure 4. The demonstrator is tied to the data acquisition shelf and makes arm movements. The sensors on the shelf convert angles into voltages through a rotational resistor. The voltage signals are filtered and stored in the memory of the data acquisition system. These data are compared with the actions generated by the actor network when the DRL algorithm is training. The reward function of the algorithm is modified to be in a faster and more stable direction by the deep neural network. Finally, the learning algorithm generates the next moment action based on the current state of the agent (robot arms), the state of the next moment, the action, and the reward. After a few hours of training (training time depends on computing power), the learning algorithm gradually converges. We recorded the trajectories of the robot arms in the simulation and sent them to the robot joints within 4 ms (the real-time communication period time of BHR) so that the robot can perform an action according to the predetermined trajectories.

Because our humanoid robot was developed first, it corresponds to a dedicated control algorithm, and its mechanical structure and motor characteristics do not allow the collection of demonstration data using the robotic arm as in some of the abovementioned studies. We thus developed a system to acquire data on robot’s joints. The idea is to conveniently and quickly capture the general trajectory of the joints of a human limb in motion and provide expert experience for network training to solve the problem of a cold start. The advantages of this method are as follows: (1) Low cost. The entire body of the rotary potentiometer and the integrated circuit board account for 80% of the total cost-a few hundred US dollars. (2) Versatility. Humanoid robots controlled by the DRL can be used to extract demonstration data using the data acquisition system designed here. Given the little variation in the physical dimensions of Asian people, the influence of the length of the upper and the lower arm on the results was negligibly small.

#### 3.2.1. Design of Data Acquisition Shelf

**Figure 5:**
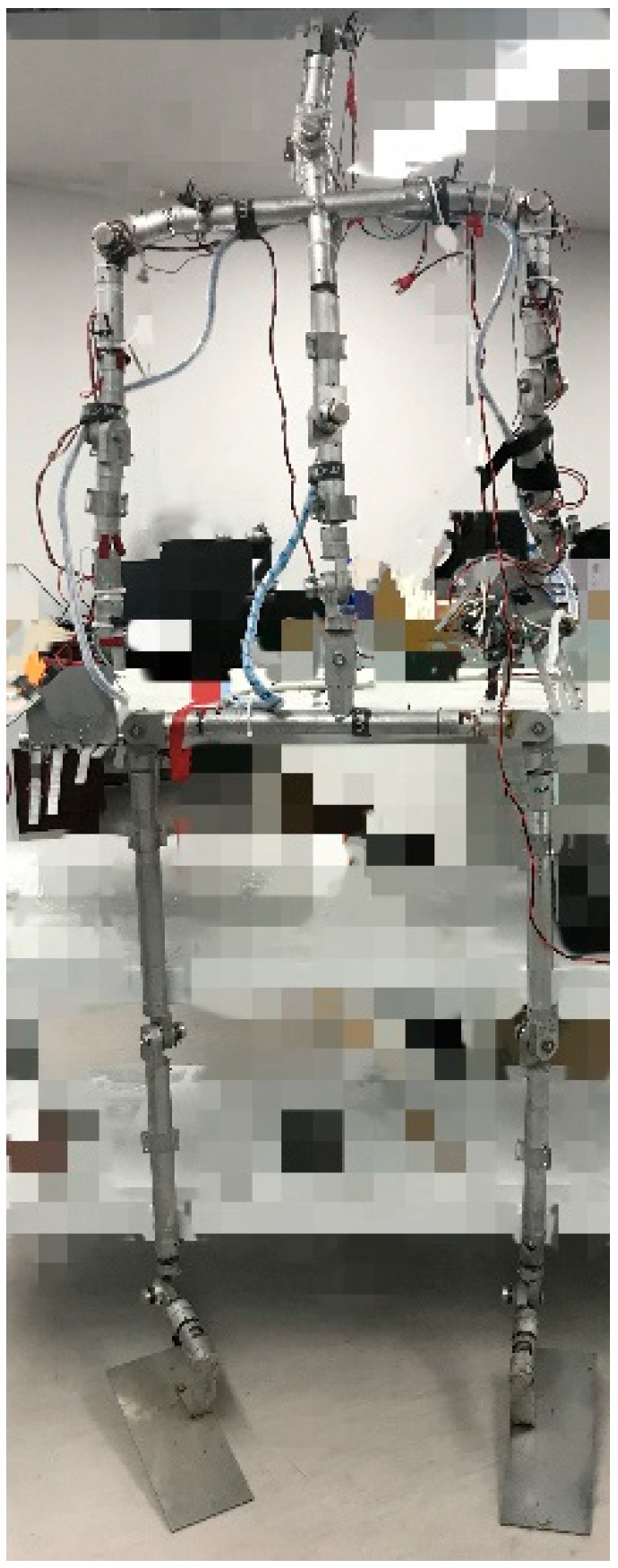
Data acquisition system.

To collect data of the humanoid robot’s arms to accelerate the training process of the DDPG algorithm, we designed the data acquisition shelf shown in Figure 5.

**Figure 6:**
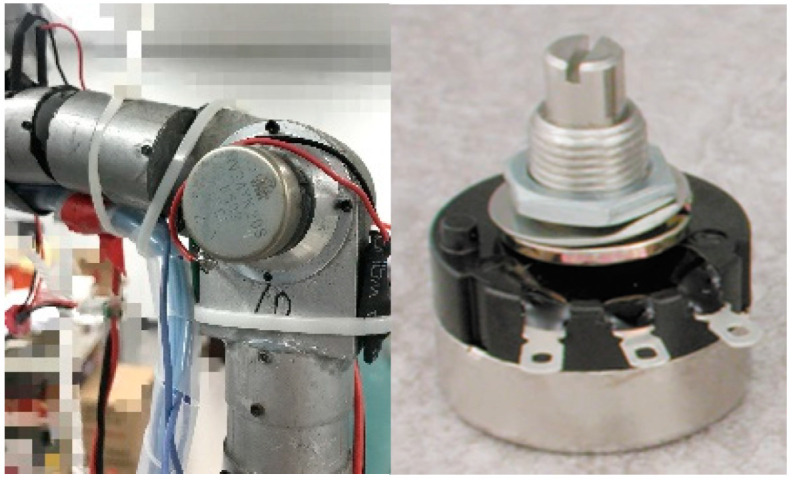
Photographs of the joint potentiometer.

The DDPG with the TSC scheme uses the angles of human motion collected by the data acquisition shelf to accelerate the training process of the vanilla DDPG. The data acquisition system consists of two parts, namely the mechanical structure (data acquisition shelf) and the control system (data processing system). The data acquisition shelf consists of two legs, redundant arms, a torso, and a head, just like the BHR humanoid robot we want to TSC. The shelf has 20 DOFs. Because the shelf is connected to the demonstrator (i.e., the arms of the demonstrator are fixed relative to the arms of the acquisition shelf), the height and limb length of the shelf are similar to those of a human. Each joint of shelf is composed of a potentiometer, a bearing, and their shell. A partially enlarged view of the sensor installed in the joint is shown in Figure 6 (left). The main structure of the potentiometer (RV24 series, TOCOS America, Inc., Schaumburg, IL, USA) consists of a resistor, three pins, and a rotary system. A photograph of the potentiometer is shown in Figure 6 (right).

When a voltage of 5 V is applied between the two fixed pins of the resistor and the potentiometer is rotated by the upper arm, lower arm, and hand, the resistance between the middle pin and side pin, and thus the output voltage, changes.

**Figure 7:**
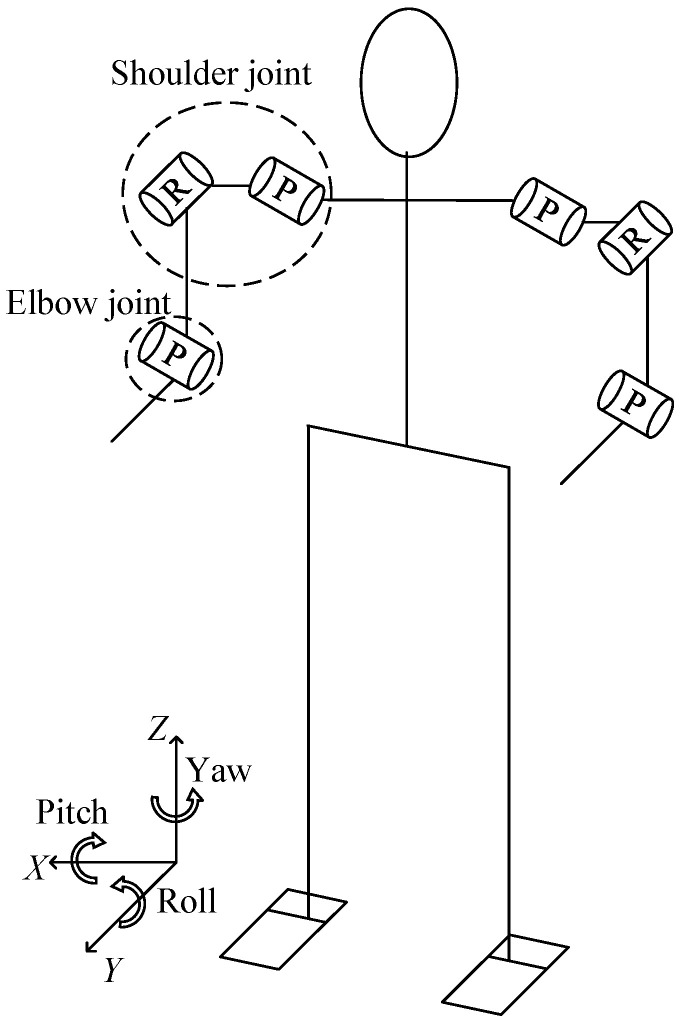
Joint configuration model for data acquisition shelf.

The humanoid TCS data acquisition shelf consist of two arms, two legs, a torso, and a neck. In our experiment, we only consider the six joints of the two arms (three DOFs for each arm); the remaining joints are fixed. We think that the simplified model, which significantly improves training efficiency, does not greatly impact the verification of the DDPG algorithm with the TCS scheme. The two joints in the shoulder are pitch and roll joints, and the joint in the elbow is a pitch joint. The other joints on the data acquisition shelf were fixed by physical means to prevent them from rotating. Motion of these joints would have coupling effects on the two DOFs of the shoulders and 1 DOF of the elbows. The joint configuration model is shown in Figure 7.

#### 3.2.2. Control System of Data Acquisition System

**Figure 8:**
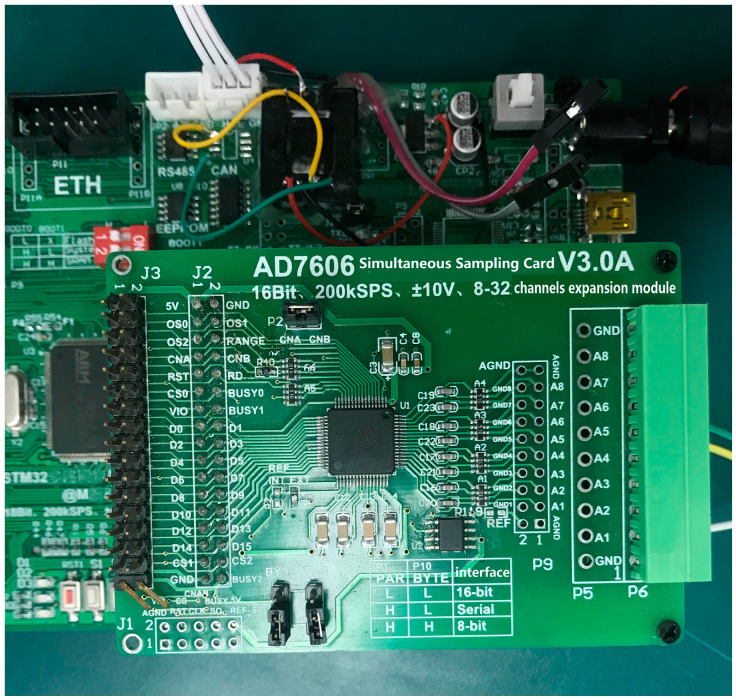
A/D acquisition module.

The output voltage of the potentiometer is analog. We used an STM32 microcontroller (STMicroelectronics, Geneva, Switzerland) as the system CPU. It takes about 5 ms to process measured data. The analog output of the potentiometer must be converted to a digital signal as the input for the STM32 microcontroller. An analog-to-digital (A/D) synchronous data acquisition module, shown in Figure 8, is used in the system. It performs A/D conversion and stores the angle data for the six joints sampled by the corresponding sensors. We determined the mapping relations between the digital STM32 input and the angle data for the six joints using zero calibration. The A/D sampling module also temporarily stores the data because the sampling rate of the STM32 is slower than the frequency of potentiometer output.

The demonstrator was tied to the data acquisition shelf and performed a series of simple actions with one arm. Because body movement is sequential, a Kalman filter is used to reduce the noise in the recorded data, such as that caused by mechanical vibrations and sensor errors. The data displayed on the upper monitor showed some errors between the measured and actual values. An analysis revealed the following three reasons for the errors:

(a) The lengths of the upper and lower arms of the data acquisition shelf were not exactly the same as those of the demonstrator.
(b) There were some calibration errors.
(c) The precision of the resistor disc deteriorated due to mechanical friction and oxidation.

**Figure 9:**
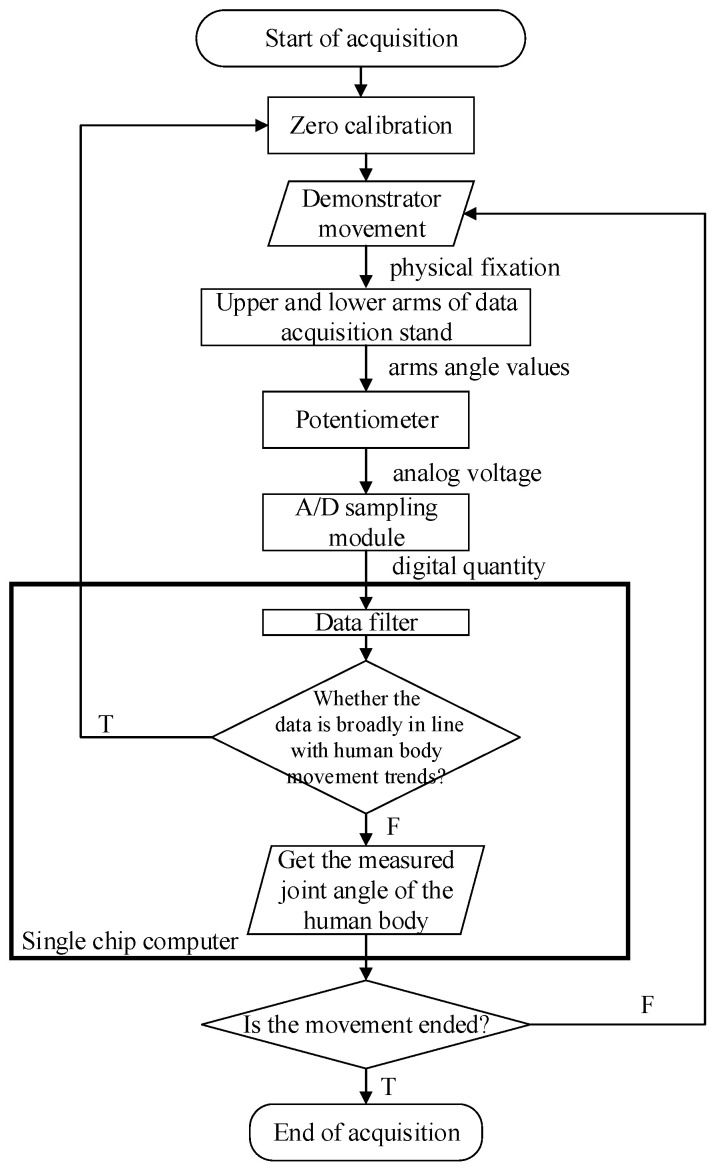
Data acquisition system workflow.

To solve these problems, the demonstrator performed a simple action and the joint angle data were recorded. We checked whether the data were broadly in line with the human body movement trends, and then recalibrated one or more sensors before recording the final human data. The zero position of the potentiometer was consistent with the initial position of the data acquisition shelf (i.e., the standing posture with arms drooping naturally). The data acquisition system workflow is shown in Figure 9.

### 3.3. Deep Deterministic Policy Gradient with TSC

We use the joint angle values of the arms of the demonstrator obtained by the data acquisition system to reproduce the reward function, enabling the DDPG to rapidly perform the training process without decreasing the accuracy of the model.

#### 3.3.1. DDPG Algorithm

**Figure 10:**
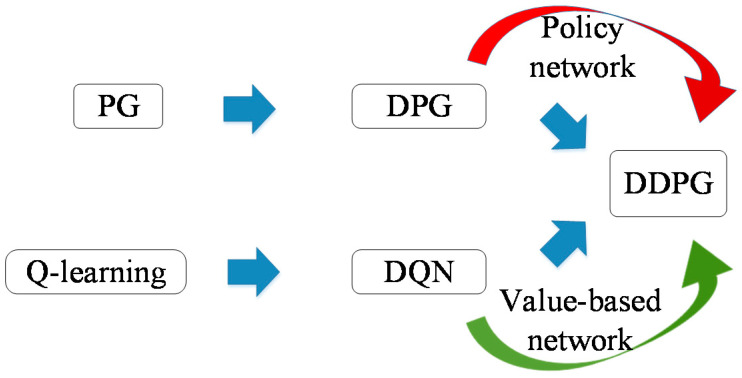
Vanilla DDPG.

The DDPG combines elements of value-function- and policy-gradient-based algorithms. With a deep neural network, following the actor-critic architecture, *μ*(*s*|*θ^μ^*) is a parameterized actor function maintained by DPG, and the critic *Q*(*s,a*) is learned from Q-learning to use the Bellman equation. The vanilla DDPG is shown is Figure 10.

The policy gradient (PG) [46] algorithm integrate exploration and improvement into a stochastic policy *π_θ_*(*a*|*s*) (usually a Gaussian strategy), because of which the calculation is simple and the theory is mature. However, because the formula for the policy gradient pertains to the expectations of states and actions, when the expectations are solved for, the distributions of the states and actions need to be integrated, which requires a large number of samples in both spaces such that the average is approximately equal to the expectation. However, for multi-joint robots, a stochastic policy to sample a large amount of data in the action space is unfeasible owing to the large number of dimensions.

Unlike the stochastic policy, the deterministic policy *a* = *μ_θ_*(*s*) is in the same policy (*θ* is the same) and an action is uniquely determined in state *s*. Therefore, this method requires a small amount of sampled data and the algorithm is highly efficient. Because the deterministic policy cannot be used to explore the environment, off-policy methods are used. “Off” here means that the action policy (actor) and the evaluation policy (critic) are not identical. The Q-learning method TD(0) is used in the evaluation policy of the DDPG to estimate and learn the action values. The task of the actor is to enable the actor network seek the maximize the value *Q_θ_*(*s,a*) of *θ*. This neural network replaces the Monte-Carlo method of prediction method, such as the cumulative discounted return used in PG method. However, when using the DNN for function approximation, RL is often unstable because the input data are usually assumed to be independently and identically distributed during training. However, the RL data are collected sequentially and the Markov property obtains between the data items. Therefore, the DDPG algorithm introduces an experience replay buffer to the DQN and the independent target networks to break the correlation between the data.

Based on the actor-critic framework with a chain rule, the actor is updated by Equation (1). DDPG uses *Q*(*s,a*) as a critic to evaluate the policy *μ*(*s*|*θ^μ^*). The cumulated reward is defined as the sum of the discounted future reward $R_{t}=\sum_{i=t}^{T}\gamma^{\left(1-t\right)}r\left(s_{i},a_{i}\right)$ with discounting factor *γ* ∈ [0,1]:

(1) $$\begin{array}{c} \;\;\;\nabla_{\theta^{\mu }}J\approx E_{s_{t}\sim \rho^{\beta }}[\nabla_{\theta^{\mu }}Q\left(s,a|\theta^{Q}\right){|}_{s=s_{t},a=\mu \left(s_{t}|\theta^{\mu }\right)}]\\ =E_{s_{t}\sim \rho^{\beta }}[\nabla_{a}Q\left(s,a|\theta^{Q}\right){|}_{s=s_{t},a=\mu \left(s_{t}\right)}\nabla_{\theta^{\mu }}\mu \left(s|\theta^{\mu }\right){|}_{s=s_{t}}] \end{array}$$

where *β* is a stochastic behavior policy different from *μ*. Since the deterministic policy is used by DDPG, and the value function does not depend on any policy. DDPG does not need to perform importance sampling like a stochastic policy. The off-policy method exploratory strategy is used by the agent to generate data, and the gradient of policy is calculated based on the data. So assuming that the samples comes from the policy *ρ^β^*. *θ^μ^* is a parameter that generates the actor network and *θ^Q^* is that for a critic network. The critic network is trained by minimizing the loss:

(2) $$L\left(\theta^{Q}\right)=E_{s_{t}\sim \rho^{\beta },a_{t}\sim \beta ,r_{t}\sim E}\left[{\left(Q\left(s_{t},a_{t}|\theta^{Q}\right)-y_{t}\right)}^{2}\right]$$

where:

(3) $$y_{t}=r\left(s_{t},a_{t}\right)+\gamma Q^{\prime }\left(s_{t+1},\mu^{\prime }\left(s_{t+1}|\theta^{\mu^{\prime }}\right)|\theta^{Q^{\prime }}\right)$$

To balance exploration and exploitation, an exploration policy *μ’* is added to the noise sampled from a noise process *N* to the actor policy:

(4) $$\mu^{\prime }\left(s_{t}\right)=\mu \left(s_{t}|\theta_{t}^{\mu }\right)+Ν$$

where *N* is usually used for Ornstein-Uhlenbeck (OU) noise due to the inertia in physical control problems, defined in Equation (5). *θ* > 0 and *σ > 0* are parameters and *W_t_* denotes the Wiener process. The OU process is a sequential process that is used to implement reinforcement learning exploration in DDPG:

(5) $$dx_{t}=\theta \left(\mu -x_{t}\right)dt+\sigma dW_{t}$$

The above is different from Gaussian noise commonly used in reinforcement learning because OU noise is suitable for inertial systems, especially when the granularity of time discretization is low-such as in the environment in which the robot operates in this study, with control period of tens of milliseconds. According to Equation (5), it can be noted that it belongs to mean regression. It is often not as different as the Gaussian noise between two adjacent, but it explores a certain distance along the positive or negative direction near the mean, which is conducive to exploration in one direction.

#### 3.3.2. Design of Reward Function

Based on the DDPG algorithm described in Section 3.3.1 and the angle data recorded from the data acquisition system, we established the following representation of the reward function:

(6a) $$\;\;\;\;\;\;\;\;r_{p}=-\lambda \ln\left[\frac{1}{n}\overset{n}{\underset{i=1}{\sum }}\eta_{i}{\left(\theta_{i}-{\hat{\theta }}_{i}\right)}^{2}\right]$$

(6b) $$r_{p}=-\lambda \ln\left[\frac{1}{n}\overset{n}{\underset{i=1}{\sum }}\eta_{i}\left|\theta_{i}-{\hat{\theta }}_{i}\right|\right]$$

where *λ* is an empirical coefficient that we set to a small number. Equation (6a) generates the value of *r_p_* for Experiment 1 and Equation (6b) that for Experiment 2. The values of all angles and the recorded values of the TSC system at this time are used to average the sum of squared errors, where the weights are different. The natural logarithm is introduced because the differences in angle and rewards follow a nonlinear logarithmic relationship. According to the angle measured by the rotary potentiometer of the data acquisition shelf, there was an error between the angle of rotation of the human joint corresponding to the movement of the demonstrator. To reduce the impact of errors on training, we used logarithmic transformation. When the cumulative value of the angle of joint output by the network is not considerably different from that of the input demonstration data, *r_p_* increases rapidly without the need for precise equality. This speeds up the training process. A certain error is also obtained between angles of the body and shelf itself (according to our measurements, the maximum error was no more than five degrees).

This reward function was designed in light of a large number of experiments after we had synthesized a variety of programs. *i* is the number of DOFs of the joints. *θ_i_* is the accumulation value of action of the actor network output, shown in Equation (7), and ${\hat{\theta }}_{i}$ is that for the collection of our data acquisition system, where *t* is the maximum number of iterations per training episode:

(7) $$\theta_{i}=\overset{t}{\underset{t=1}{\sum }}a_{it}$$

Although the agent we designed for DDPG can only perform motions similar to those of a conventional robot, we hope that each joint can move like a humanoid robot or even a person as different joints have different performance. *η_i_* is a parameter it indicates the level at which different joints learn from the data acquisition system. In the simulation, we multiply the action of each joint calculated by the neural network by several coefficients *η_i_*. This represents the expectation of the learning intensity of each joint. *η_i_* must be set separately at the beginning of training. If robot joint *i* requires high precision, we can set its *η* to a large value. In this case, a smaller reward of prediction (*r_p_*) will be obtained in the initial stage of training. A bigger *η* can punish those joints that do not match the input from the data acquisition system, but the training time will be increased. If we want to quickly develop robot movement without having to deal with the joint execution errors of joint *i*, a smaller learning rate can be chosen to make *r_p_* larger at the beginning of training. We can set different learning rates for different joints, which have different mechanical structures.

**Figure 11:**
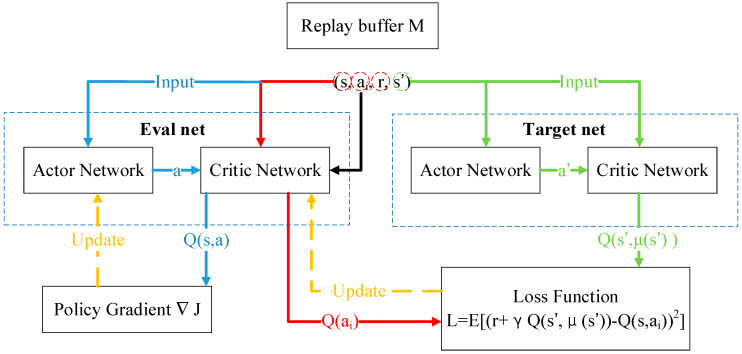
Actor-critic structure for DDPG with TSC.

In the early stages of network training, *θ_i_* is very different from the demonstrated joint angle ${\hat{\theta }}_{i}$ and *r_p_* is a small negative number. When *θ_i_* and ${\hat{\theta }}_{i}$ are equal to 1°, which is the case in the final stage of training, *r_p_* = 0. When the average difference is less than 1°, the reward *r_p_* rises sharply. Algorithm cheating (i.e., one joint is extremely accurate but another joint is not) is not a concern. Because the natural logarithm function is nonlinear, as long as the search space is large enough and the learning rate is appropriate, this part of the reward function can increase rapidly when the difference is 1° or less. Therefore, *r_p_* can be considered as a dynamic compensation factor that adjusts the reward function *R* of DRL in real time. The components of *R* depend on the learning task. *r_p_* is an essential part of the TSC scheme. Figure 11 shows the data transfer between the four neural networks in the actor-critic structure, where each group (*s,a_i_,R,s′*) comes from the replay buffer *M*.

The DDPG with TSC is described in Algorithm 1.

**Algorithm 1** DDPG with TSCRandomly initialize critic network *Q*(*s,a*|*θ^Q^*) and actor *μ*(*s*|*θ_μ_*) with weights *θ^Q^* and *θ^μ^*. Initialize target network *Q′* and *μ′* with weights $\theta^{Q^{\prime }}\leftarrow \theta^{Q},\;\theta^{\mu^{\prime }}\leftarrow \theta^{\mu }$ Initialize replay buffer *M* Import a group of joint angle ${\hat{\theta }}_{i}$ recorded by TSC Generate posterior estimates from Kalman filter **for**
*ep* = 1, *EPISODE*
**do** Initialize a random process *χ* for action exploration Get initial state *s*_1_ **for**
*t* = 1, *STEP*
**do**  Select action *a_t_* = *μ*(*s_t_*|*θ^μ^*) + *N_t_* according to current policy and exploration noise  Execute *a_t_* to get reward rt and update the joint angle value *r_t_* and update the joint angle value (*θ*_1*t*_,*θ*_2*t*_,…*θ_nt_*), and observe new state *s*_*t*+1_.  Calculate the *r_p_* with (*θ*_1*t*_,*θ*_2*t*_,…*θ_nt_*) and (${\hat{\theta }}_{1t},{\hat{\theta }}_{2t},\ldots ,{\hat{\theta }}_{nt}$):$$r_{p}=-\lambda \ln\left[\frac{1}{n}\overset{n}{\underset{i=1}{\sum }}\eta_{i}{\left(\theta_{it}-{\hat{\theta }}_{it}\right)}^{2}\right]$$  Add *r_p_* to reward *r*:$$R\;=\;r\;+\;r_{p}$$  Store transition (*s_t_*,*a_t_*,*R_t_*,*S*_*t*+1_) in *M*  Sample a random mini-batch of *N* transitions (*s_t_*,*a_t_*,*R_t_*,*S*_*t*+1_) from *M*  Calculate *y_i_* with critic target network and actor target network  Update critic by minimizing the loss: $L=\frac{1}{N}\sum_{i}\left(y_{i}-Q(s_{i},a_{i}|\theta^{Q}\right){)}^{2}$  Update actor policy using the sampled policy gradient:$$\nabla_{\theta^{\mu }}J\approx \frac{1}{N}\underset{i}{\sum }\nabla_{a}Q\left(s,a|\theta^{Q}\right){|}_{s=s_{i},a=\mu \left(s_{i}\right)},\nabla_{\theta^{\mu }}\mu (s|\theta^{\mu }){|}_{s_{i}}$$  Update the target networks:$$\theta^{Q^{\prime }}\leftarrow \tau \theta^{Q}+\left(1-\tau \right)\theta^{Q^{\prime }}$$$$\theta^{\mu^{\prime }}\leftarrow \tau \theta^{\mu }+\left(1-\tau \right)\theta^{\mu^{\prime }}$$ end for end for

## 4. Implementation

We designed two experiments to verify the performance of the proposed scheme for a humanoid robotic arm that is used for rapid planning of multi-task as DRC mentioned in Section 1. The first experiment was implemented in Python 3.7 and the second experiment was implemented in a co-simulation in Python and V-REP. V-REP is a robot simulator with an IDE and based on a distributed control architecture. We used the remote API for communication, with a Python IDE as the client and V-REP as the server. We verified the effectiveness of our DDPG with the TSC scheme in terms of stability and speed. We then designed the vanilla DDPG experiment as a control group compared to our algorithm improved from TSC scheme.

### 4.1. Trajectory Planning for a Planar 3-DOF Robotic Arm

The planar 3-DOF robotic arm was trained by the DDPG with the TSC scheme, which guided the fingers to perform motion with the desired trajectories. To simplify the simulation, we used an arc as a motion trajectory task to be learned. The arm was composed of three segments, namely the upper arm *OA*, the lower arm *AB*, and the hand *BC*, all in a 2D plane. *O*, *A* and *B* are three joints, and thus *n* = 3. The directions of the joint axes are in the vertical window facing inward, respectively, like the human shoulder, elbow, and wrist. Because the experiment was performed in a virtual 2D simulation environment, the joint angle data could not be obtained with the data acquisition shelf. Instead, a start point, an end point, and a few (~5) intermediate points of the arm trajectory were chosen, and then the B-spline interpolation algorithm was used to obtain a trajectory curve, which was used as the finger trajectory of a human demonstrator. For convenience of programming, 50 sample points on the curve generated by B-spline interpolation were extracted as the target points that the robot arms needed to learn to reach. $T=\left(T_{1},T_{2},\cdots T_{K-1},T_{K}\right)$, where *K* = 50, are the center points of the moving target trajectory in the simulation environment. The movement from *T_1_* to *T_50_* represents the trajectory of the target points. The training goal of the learning algorithm is to make the fingertip *C* continuously track the movement of *T_i_*, where *i* = 1 to 50, so that the fingertip *C* can move according to the desired arc trajectory.

**Figure 12:**
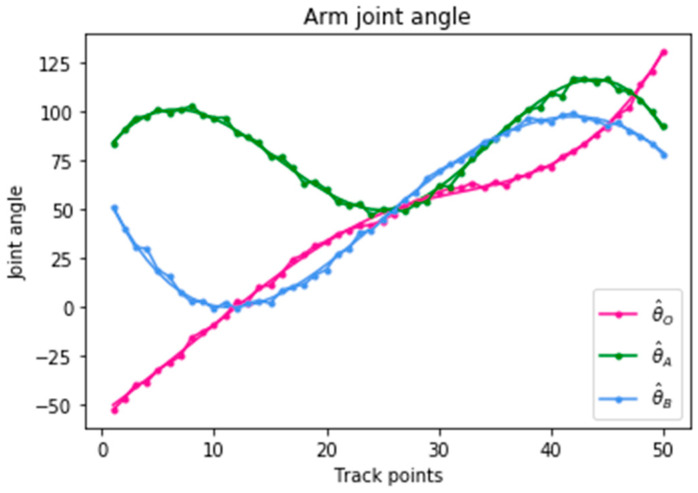
Arm joint angle with Gaussian noise.

**Figure 13:**
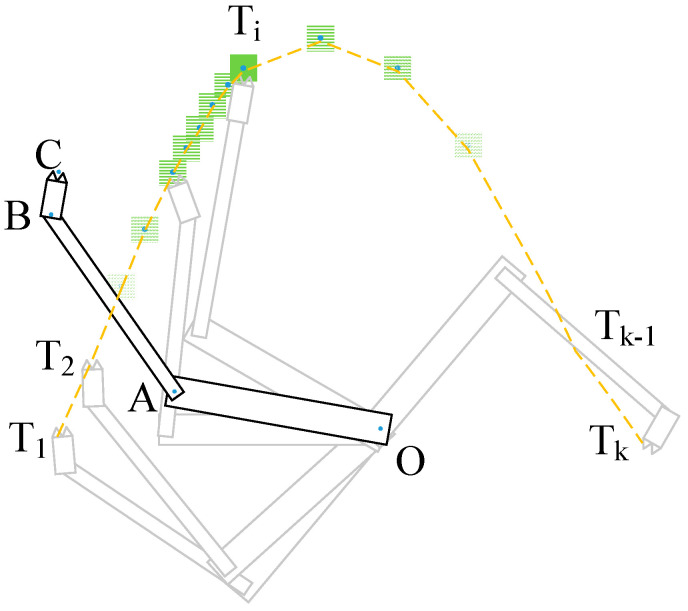
3-DOF arm movement trajectory.

We simulated the movement of the robotic arm according to the fingertip motion trajectory curve, and obtained a set of three joint angle trajectory curves, as shown by the three solid lines ${\hat{\theta }}_{O}$, ${\hat{\theta }}_{A}$, and ${\hat{\theta }}_{B}$. Due to space limitations, Table 1 shows only some of the data of TSC generated by the B-spline interpolation algorithm. Gaussian noise with μ=0 and $\sigma^{2}=2$ was added to the three joints when they passed each trajectory point, as shown by the dots on each solid line in Figure 12, with some slight jitter. The approximate movement trajectory is shown in Figure 13.

When a DRL agent is trained at each step of a track point, the reward function *R* is obtained, as shown in Equation (8); it consists of three parts, namely *r_o_*, *r_d_*, and *r_p_*. Reward *r_o_* is derived from the distance from fingertip *C* to target *T*. It is the most significant part of the reward function, as it reflects the accuracy of the fingertips of the robot arm. *r_d_* is related to the duration time of *C* in contact with target *T*; it reflects the stability of the arm after it reaches the target. *r_p_* is derived from the TSC scheme. Although our goal is to make the fingertip *C* touch *T_i_* rapidly and stably, *r_d_* increases as fingertip *C* stays in the green box continues to grow as the trajectory shown in Figure 13, the distance of other joints relative to the target still needs to be used. The trajectory is obtained from the interpolation points, which we call track points:

(8) $$R=r_{o}+r_{d}+r_{p}$$

In each time step *t* of track point *k* for a training episode, we compare $\left(\theta_{1tk},\theta_{2tk},\theta_{3tk}\right)$ and $\left({\hat{\theta }}_{1k},{\hat{\theta }}_{2k},{\hat{\theta }}_{3k}\right)$, where *k = 1,2,…,K* and *t = 1,2,…,T*. Here, the joint angle ${\hat{\theta }}_{ik}$ is obtained from the data acquisition system and the angle accumulation value $\theta_{itk}$ is generated by the actor network of the DDPG. The difference between $\theta_{itk}$ and ${\hat{\theta }}_{ik}$ makes the reward function less discrete; a discrete reward function hinders the convergence of the network.

**Figure 14:**
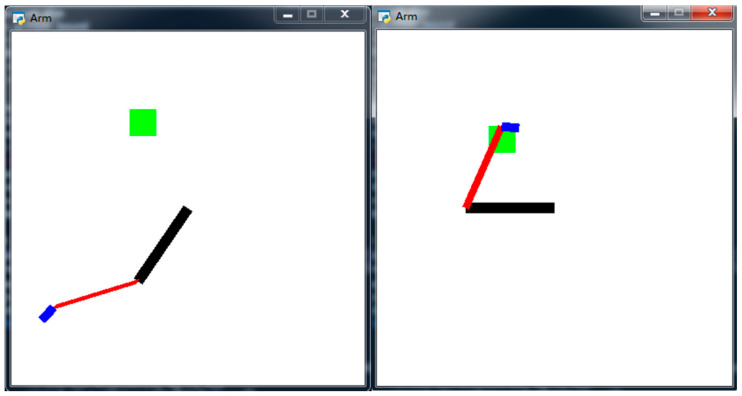
Schematic diagram of planar 3-DOF robotic arm.

**Figure 15:**
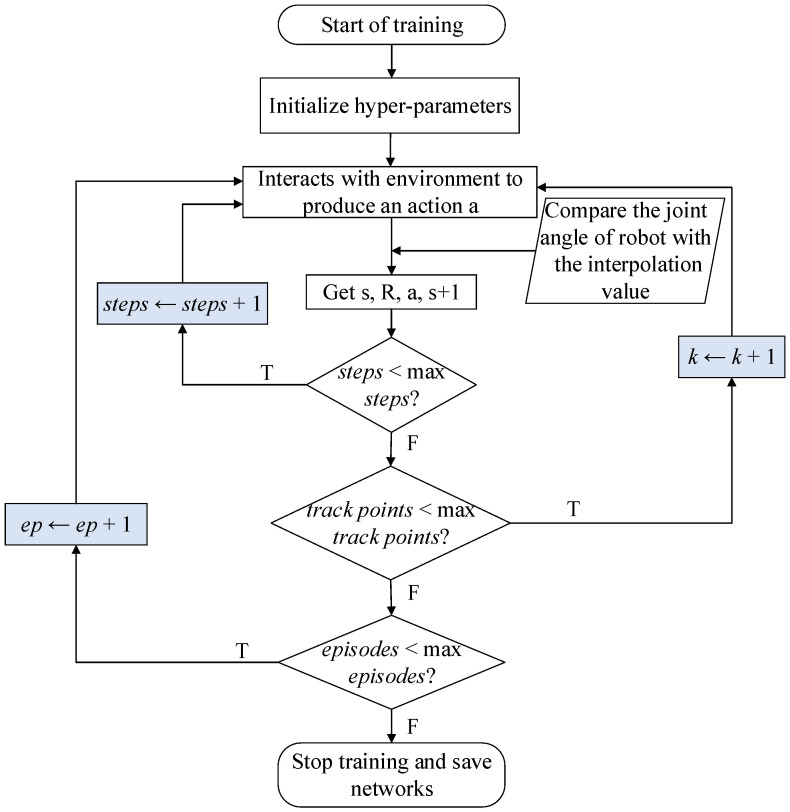
Flow chart of first experiment using DDPG with TSC.

In a certain training episode, the coordinates of joint *O* are assumed to be (*x_o_,y_o_*), which is also the initial position of the coordinates. For convenience, joint *O* can be any coordinate value in the 2D coordinate system. The coordinates are $\left(x_{A},y_{A}\right)$ for joint *A*, $\left(x_{B},y_{B}\right)$ for joint *B*, $\left(x_{C},y_{C}\right)$ for fingertip *C*, and $\left(x_{T_{i}},y_{T_{i}}\right)$ for track point *T_i_*, where *i* is a loop variable that ranges from 1 to *K*. Figure 14 shows the simulation environment when the robotic arm moves. The maximum number of steps trained at one point is 400. When the termination condition of each iteration is reached, loop variable $T_{i}\leftarrow T_{i+1}$, and the network continues to train *C* to reach the next target *T_i_*. After all track points have been trained as target points, ep←ep+1 and *i* is reset to 1. The algorithm flow chart is shown in Figure 15.

**Figure 16:**
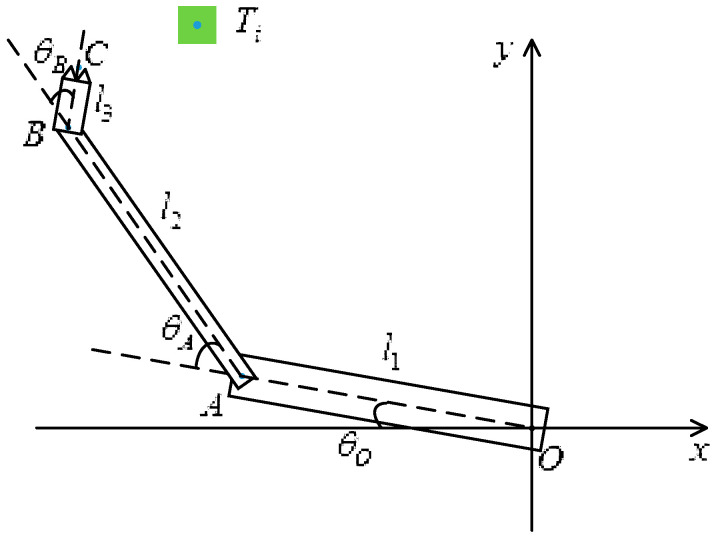
3-DOF arm model.

As shown in Figure 16, the rotation angle between the negative direction of the *x*-axis and upper arm *OA* is defined as $\theta_{O}$, that between the *OA* extension line and lower arm *AB* is defined as $\theta_{A}$, and that between the *AB* extension line and hand *BC* is defined as $\theta_{B}$. The lengths of *OA*, *AB*, and *BC* are $l_{1}$, $l_{2}$, and $l_{3}$, respectively. Therefore, the coordinates of *A*, *B*, and *C* can be derived as:

(9) $$\left(x_{A},y_{A}\right)=\left[\cos\left(180{}^{\circ}-\theta_{O}\right),\sin\theta_{O}\right]\cdot l_{1}+\left(x_{O},y_{O}\right)$$

(10) $$\left(x_{B},y_{B}\right)=\left\{\cos\left[180^{{}^{\circ}}-\left(\theta_{O}+\theta_{A}\right)\right],\sin\left(\theta_{O}+\theta_{A}\right)\right\}\cdot l_{2}+\left(x_{A},y_{A}\right)$$

(11) $$\left(x_{C},y_{C}\right)=\left\{\cos\left[180^{{}^{\circ}}-\left(\theta_{O}+\theta_{A}+\theta_{B}\right)\right],\sin\left(\theta_{O}+\theta_{A}+\theta_{B}\right)\right\}\cdot l_{3}+\left(x_{B},y_{B}\right)$$

The state S of the DDPG consists of the coordinates of O, A, B, and C, and the increment of the coordinates between pairs of points $AT_{i}$, $BT_{i}$, and $CT_{i}$. We added an indicator I at the end of the array to indicate whether the finger touches the target for a certain time. The state S of each track point is updated every step. The array of the state S is as follows:

$$s=\left[\left(x_{A},y_{A}\right),\left(x_{B},y_{B}\right),\left(x_{C},y_{C}\right),incre_{AT}+incre_{BT}+incre_{CT},I\right]$$

where:

$$incre_{AT}=\left[\left(x_{T}-x_{A}\right),\left(y_{T}-y_{A}\right)\right]$$

$$incre_{BT}=\left[\left(x_{T}-x_{B}\right),\left(y_{T}-y_{B}\right)\right]$$

$$incre_{CT}=\left[\left(x_{T}-x_{C}\right),\left(y_{T}-y_{C}\right)\right]$$

According to Equations (6) and (7), we expand $r_{P}$ as:

(12) $$r_{P}=-\lambda \ln\left\{\frac{1}{3}\left[\eta_{O}{\left(\theta_{O}-{\hat{\theta }}_{O}\right)}^{2}+\eta_{A}{\left(\theta_{A}-{\hat{\theta }}_{A}\right)}^{2}+\eta_{B}{\left(\theta_{B}-{\hat{\theta }}_{B}\right)}^{2}\right]\right\}$$

The hyperparameters of the model are listed in Table 2.

For the first experiment, the network structure of the actor was as follows: The number of neurons in the input layer was 13 (the number of dimensions of the state feature was 13), the middle layer was a fully connected layer with 600 neurons, and the activation function was the ReLU. To limit the action of the output to a certain range, the output layer used tanh as the activation function of the fully connected layer. The output was a deterministic policy that was multiplied by a boundary value as the output of the actor network. The structure of the critic network was similar to that of the actor network, but the output layer generated the value of Q and did not use an activation function. Both networks had two neural networks, a target network and a main network. Therefore, the entire algorithm consisted of four DNNs. The second experiment had the same network structure as the first, except that the numbers of neurons in the output and the middle layers of the neural network were different.

**Algorithm 2** First experimentInitialize hyper-parameters of DDPG with TSC scheme Select 50 track points and use B-spline interpolation to obtain joint trajectory curves of three joints Import ${\hat{\theta }}_{i}$ using B-spline interpolation algorithm **for**
ep = 1, EPISODE
**do** Initialize a random process χ for action exploration Get initial state $S_{1}$ **for**
k = 1, max track points K do Update arm target point $T_{k}$  **for**
t = 1, STEP
**do**   Select and execute action $a_{t}$ to get $r_{o}$, $r_{d}$, $r_{t+1}$, and update the joint angle value ($\theta_{1t},\theta_{2t},\cdots ,\theta_{nt}$).   Calculate the $r_{p}$ with ($\theta_{1t},\theta_{2t},\cdots ,\theta_{nt}$) and (${\hat{\theta }}_{1t},{\hat{\theta }}_{2t},\cdots ,{\hat{\theta }}_{nt}$):$$r_{P}=-\lambda \ln\left[\frac{1}{n}\overset{n}{\underset{i=1}{\sum }}\eta_{i}{\left(\theta_{it}-{\hat{\theta }}_{it}\right)}^{2}\right]$$   Get total reward R:$$R=r_{o}+r_{d}+r_{p}$$   Store ($s_{t},a_{t},R_{t},s_{t+1}$) in M and sample from M   Calculate $y_{i}$ with critic target network and actor target network   Update actor and critic network on-line networks by L and $\nabla_{\theta^{\mu }}J$   Update the target networks by soft-replacement parameter τ  end for end for end for

### 4.2. Humanoid Robot Arm Simulation

To verify the algorithm, we planned the driving trajectory of a steering wheel for the robot arm in the simulation environment. One task in the DRC was for a robot to drive a vehicle, which was slightly modified but still retained a traditional steering mechanism, to a designated location. Inspired by this task, we used DRL to make the robot learn how to turn a steering wheel based on the required steering wheel angle. In addition, the palms of the robot were not fixed on the steering wheel to allow the robot’s actions before and after driving the vehicle to be linked without the need for manual binding operations.

Table 3 shows the range constraints of the joints of the humanoid robot BHR in this simulation. These limits are set by us and completely satisfied by our experimental requirements. The algorithm search space is small enough for convergence. Due to the mechanical interference between the arms and the upper body of the robot, the rotation angle of the shoulder roll joint inward is very small (about 5°). Another important reason for limiting the angles of rotation is the limited computing power of the personal computer used for training the DDPG. The input of the network is six joint rotation angles with a continuous state-action space. Therefore, the arms can only perform up and down movements in a small range (not a circle). The position directions of Joint 1 and Joint 3 are set to the directions in which the arms rotate downwards. In other words, they follow the right-hand rule. Horizontal left is set to the positive direction of the *y*-axis. It is worth mentioning that for convenience of programming, we set the rotation direction of Joint 2 of both arms to a positive direction when movement was away from the robot’s trunk. For historical reasons, we did not correct this mistake. All joints and the steering wheel are initialized to 0°.

**Figure 17:**
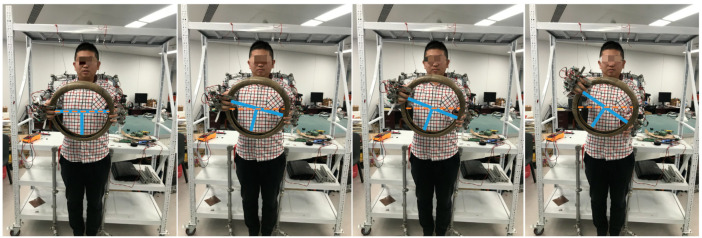
Collection of data during performance of an action.

When the demonstrator is tied to the data acquisition shelf, the three joint positions of each human arm should try to match the corresponding positions on the shelf as much as possible, as shown in Figure 17. Extreme accuracy is unnecessary because we need to obtain only a rough trajectory of an action as a guide. Specific robot joint motion planning is derived from the learning algorithm with a stochastic component.

**Figure 18:**
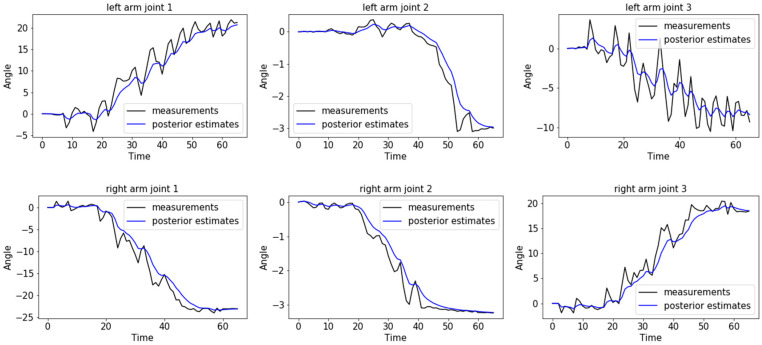
Angle values from data acquisition system.

Figure 18 shows the rotation angle values of the arm joints obtained when the demonstrator rotated the steering wheel counterclockwise by 30° to the left. Fluctuations in measurement data due to mechanical vibrations and sensor errors were recorded, as shown by the black lines. A Kalman filter was applied to the data; the obtained posterior estimate curves are shown as the blue lines. The black lines fluctuate whereas the blue lines are comparatively smooth (i.e., they have less error).

**Figure 19:**
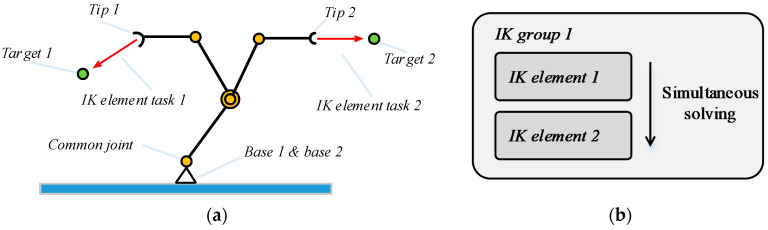
Constrained inverse kinematics solution process. (**a**) Constrained kinematic chain and (**b**) solution flow chart.

The rotation of the steering wheel is subject to inverse kinematic constraints from both arms of the robot. Figure 19 shows the workflow of obtaining the numerical solution of inverse kinematics in V-REP. A joint is shared by two kinematic chains. If kinematic chain 1 is solved first, *Tip 1* moves to the left with the joint, which affects the solution of kinematic chain 2. If kinematic chain 2 is solved first, *Tip 2* takes the joint to the right and affects the solution of IK element task 1. We can simultaneously perform the inverse solution calculation by placing two IK elements in the same IK group.

**Figure 20:**
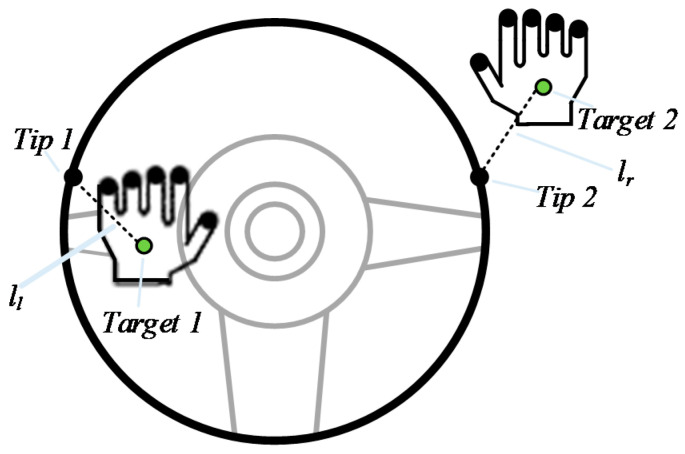
Diagram of palms on steering wheel.

The above solution of the inverse kinematics of the two arms and the steering wheel in the simulation environment is applied in this experiment. In the simulation, we placed two points on the steering wheel, which are in contact with the two palms, respectively, called *Tip 1* and *Tip 2*. The two palms are placed at two points, called *Target 1* and *Target 2*. At the beginning of each training episode of the DDPG with TSC, the distance between *Tip 1* and *Target 1* is $l_{l}$, and that between *Tip 2* and *Target 2* is $l_{r}$, both of which are 0. When the simulation starts, two inverse kinematic tasks are solved simultaneously. The values of $l_{l}$ and $l_{r}$ are obtained once per training step. The hands are placed on the steering wheel as shown in Figure 20. Although the positions of the two hands move and the steering wheel is rotated at each step, the tip and target points are fixed relative to the steering wheel and the palms, respectively.

The goal of this simulation experiment is to teach the robot how to make the steering wheel turn 30° to the left when the six joints of the robot’s arm include the steering wheel angle from the initial position of 0° at the start of the training process. The angle data of the six joints of the arms are recorded when the steering wheel is turned, which will be extracted from the expectations of the previous training episodes. Next, the data are transmitted to the humanoid robot BHR via the controller area network (CAN) communication protocol with a control period of 4 ms. The robot then performs the corresponding action in the simulation. However, this part of the content is not the focus of this study due to some specific robot control technology; further research is required.

We created this simulation environment for quickly planning trajectories of all joints for multiple tasks, but one or both hands cannot be fixed on the steering wheel even if this operation can significantly reduce the DRL calculation time and improve calculation accuracy. With such an operation, the simulation cannot quickly deploy the robot to an environment with multiple tasks. In this open environment, the robot arm can be freely moved in space without being bound by a specific task, such as rotating the steering wheel, and is only constrained by the motor rotation angle shown in Table 3. This is done to avoid an unnecessary waste of time and power when searching the joint space. When a developer wants to plan the joint trajectory of the next action of the robot arm, it is only necessary to transform the action required by the robot into a reward function in the simulation; the training process can be started immediately. When the demonstrator is connected to the data acquisition shelf and makes the corresponding actions, performance is greatly improved:

(13) $$R=r_{1}+r_{2}+r_{3}$$

(14) $$r_{1}=-\alpha \left|\theta_{s}-\theta_{t}\right|$$

(15) $$r_{2}=-\beta \ln\left[100\times \left(l_{l}+l_{r}\right)\right]$$

(16) $$r_{P}=-\lambda \ln\left(\frac{1}{n}\overset{n}{\underset{i=1}{\sum }}\left|\theta_{i}-{\hat{\theta }}_{i}\right|\right)$$

According to the analysis in Section 3.3.2, the reward function is defined as Equations (13)–(16). For convenience of description, $r_{P}$ in Section 3.3.2 is rewritten here as $r_{3}$. The reward function consists of three parts. The first part is the difference between the current angle of the steering wheel and the target angle, which is 30° from start to finish. The second part is the distance between the palms of the two hands and the steering wheel in 3D space. This part indicates whether the hand can accurately track the trajectory required to rotate the steering wheel without relative constraints between the two hands and the steering wheel. The third part is the function of the difference between the action generated by the deep networks and that collected from the TSC system. We record the data acquisition shelf according to the step size set in the simulation.

The experimental hyperparameters for the robot driving are listed in Table 4. Because we applied the DDPG with the TSC scheme to the experiments in Section 4.1 and Section 4.2, the network structure and the hyperparameters are similar, except for their values.

## 5. Results

The learning process takes place for a fixed number of time steps. To evaluate the performance of the algorithm, several simulations were performed in the first experiment. The average accumulated reward and average evolution steps of several trials better reflect the efficiency and reliability of the algorithm. The experiments were performed using a personal computer with an Intel Core i5 2520 2.5-GHz CPU and 8 GB of RAM running Windows 7. Python 3.7 was used as the programming language, TensorFlow 1.14 was used as the deep learning framework, and V-REP 3.5 EDU was used as the simulation environment in the second experiment.

### 5.1. First Experiment

In the first experiment, we performed five simulations using the DDPG with and without TSC, respectively. All results in this section are the averages from the five simulations. The goal of this experiment was to teach a controller. When the initial position *O* and the end position $T_{i}$ of the robotic arm are given, the controller can plan a trajectory according to the approximate trajectory provided by the demonstrator.

At the beginning of the training, the robotic arm could not touch the target quickly. With increasing number of training episodes, the finger gradually reached the target point, but was slightly unstable. By the end of training, the vibration of the arm disappeared; the arm could move quickly and stably to reach every point *T*.

**Figure 21:**
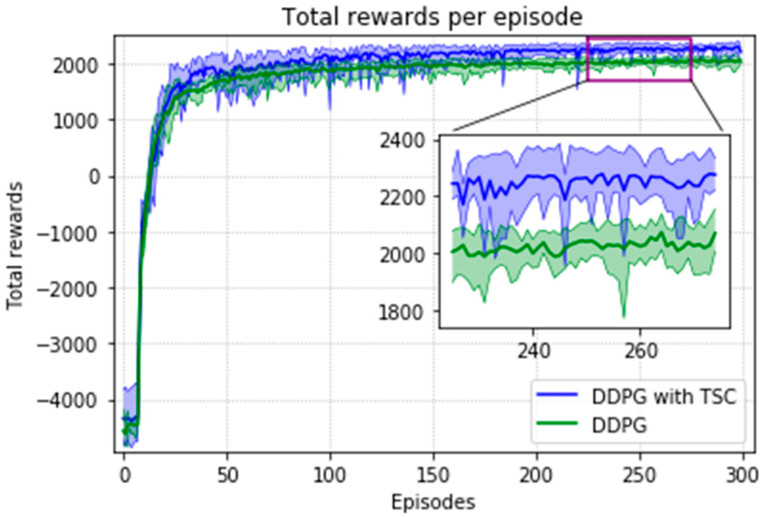
Accumulated rewards for the DDPG with and without TSC in the first experiment.

**Figure 22:**
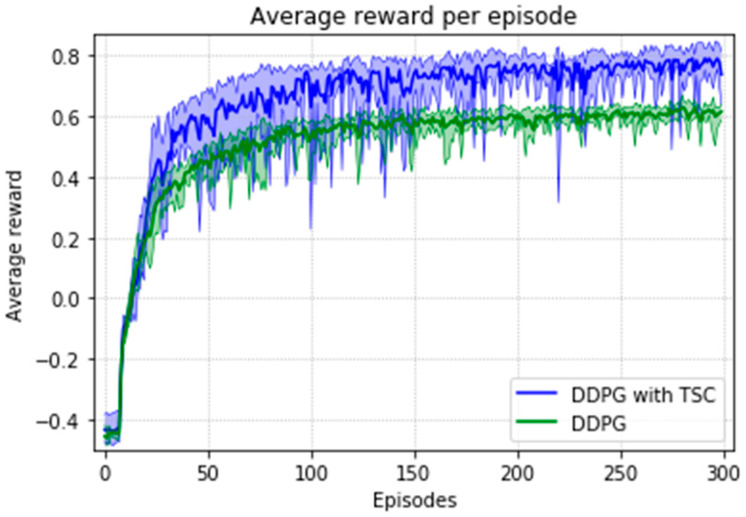
Average reward per training episode for the DDPG with and without TSC in the first experiment.

**Figure 23:**
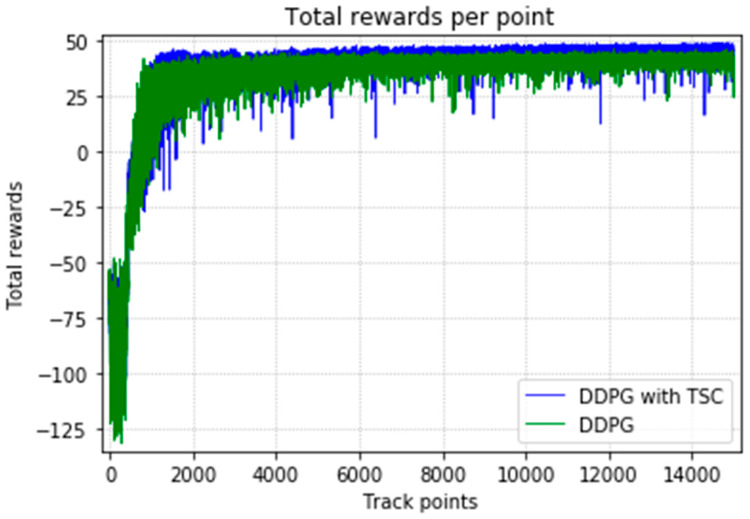
Total rewards per point for the DDPG with and without TSC in the first experiment.

**Figure 24:**
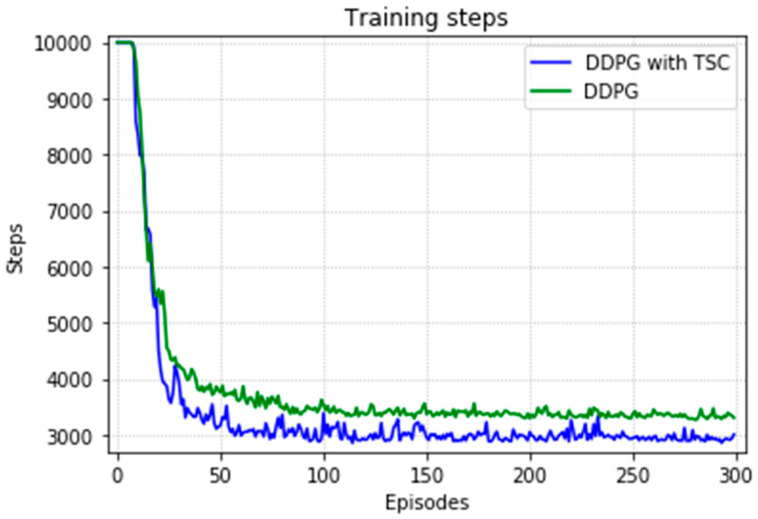
Training steps per training episode for the DDPG with and without TSC in the first experiment.

The arm was more accurate and stable when the TSC scheme was used for the DDPG. The results obtained for the DDPG with and without TSC are shown in Figure 21, Figure 22, Figure 23 and Figure 24. Figure 21 shows the average accumulated rewards obtained in each of the 300 training episodes, and Figure 22 shows the average reward for each step of the 300 training episodes. To better understand the results, the maximum and minimum values are also shown in Figure 21 and Figure 22, and their curves trend should be consistent. Because we designed 50 track points as a phased goal of DRL, a total of 15,000 points were counted. Figure 23 shows the total rewards of all points for the two algorithms. The number of evolutionary steps required to achieve the goal over 300 training episodes is shown in Figure 24.

**Figure 25:**
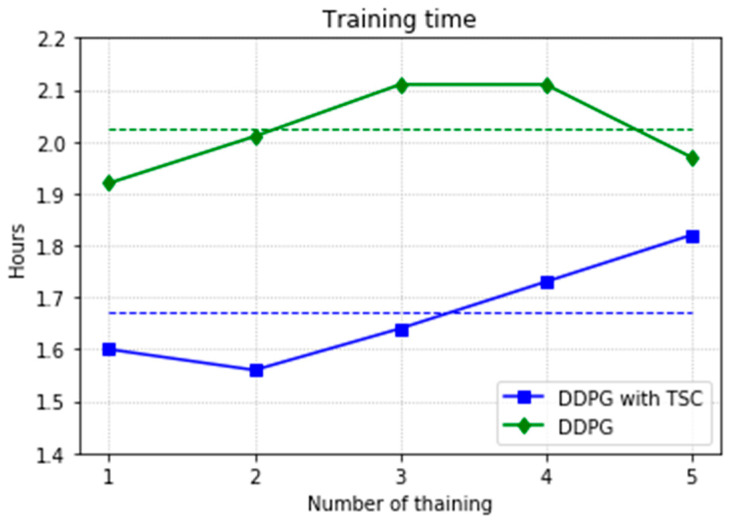
Training time for the DDPG with and without TSC in the first experiment.

Almost all performance indicators related to network training, including the training time shown in Figure 25, show that the DDPG with TSC for reward improvement is much better than the vanilla DDPG algorithm. The dotted line in the figure is the average time of five simulations corresponding to the broken line. With the imported human joint angles collected by the data acquisition system, the DDPG with TSC can significantly improve the average reward of each step. This allows the robotic arms to rapidly complete the seemingly disordered and aimless search process in the state space. At the 300th training episode, the average reward increased by 20.00%, the number of training steps decreased by 8.99%, and the average training time decreased by 17.33% compared with those for the vanilla DDPG.

**Figure 26:**
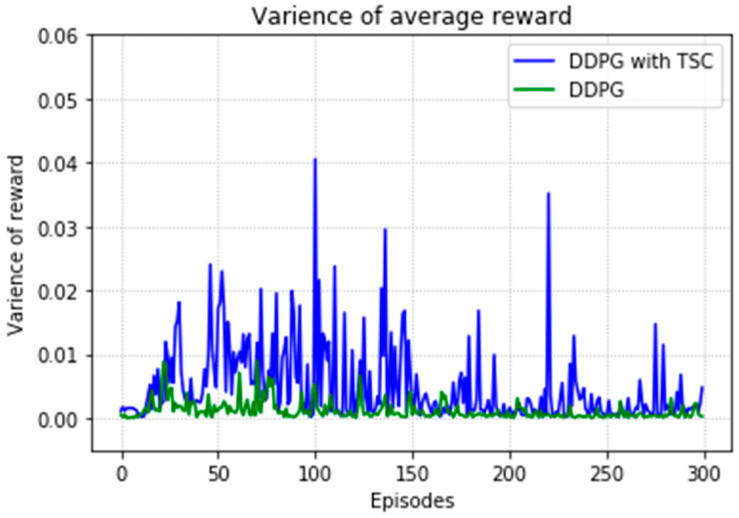
Variance of average reward for the DDPG with and without TSC in the first experiment.

**Figure 27:**
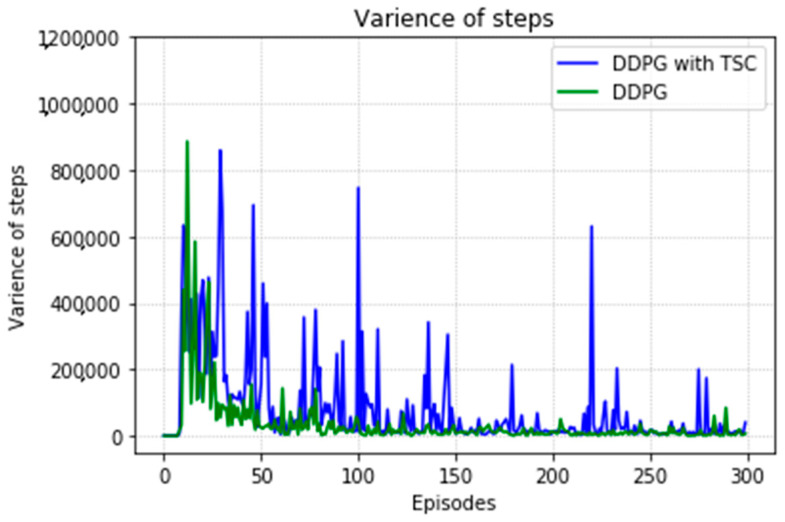
Variance of training steps for the DDPG with and without TSC in the first experiment.

Regarding the average return at each training episode, Figure 22 shows that there are several large fluctuations in the *y*-axis direction during the training process. This feature is reflected in the evolutionary steps required for each episode shown in Figure 24. This phenomenon is highlighted in the variance comparison shown in Figure 26 and Figure 27. We think that this situation is due to the combination of some of the data points (${\hat{\theta }}_{O}$, ${\hat{\theta }}_{A}$, and ${\hat{\theta }}_{B}$) and points obtained from the B-spline interpolation, which may result in the trajectory of the arm movement being unable to reach a very compliant path. Although the variance curve looks abrupt, it is surprising that the value is not very large and its effect is similar to DDPG. The disadvantage of DDPG with TSC is negligible compared with the improvement. This problem can be solved in future work.

### 5.2. Second Experiment

Based on a consideration of time consumption and computer performance limitations, we performed four tests for the TSC scheme in this experiment and four tests for the vanilla DDPG algorithm as the control group (denoted as the TSC-free scheme). It is difficult for the TSC-free scheme to find the global optimal solution in a limited time. The TSC scheme thus outperforms it in all results.

**Figure 28:**
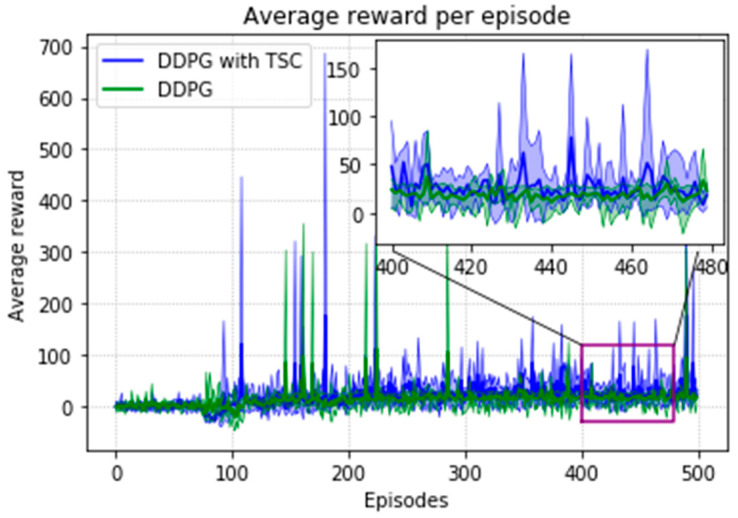
Average reward per training episode for the DDPG, with and without TSC, in the second experiment.

**Figure 29:**
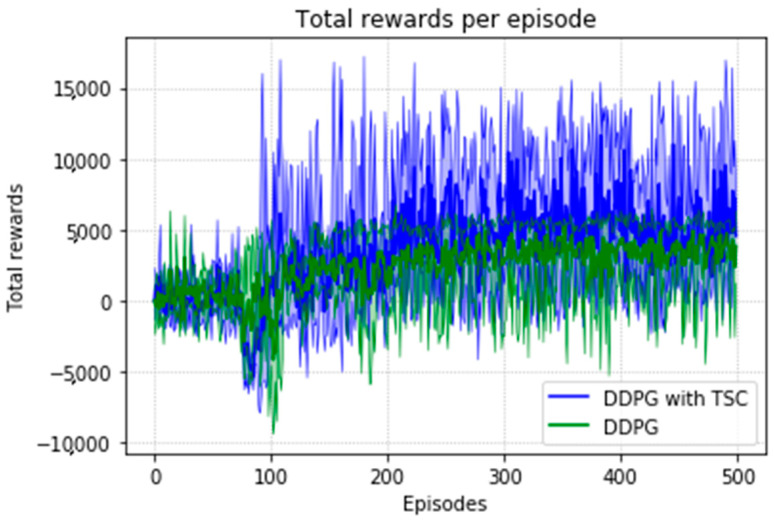
Accumulated rewards for the DDPG with and without TSC in the second experiment.

Due to the natural logarithmic function of $r_{3}$ in Equation (16), after converging to a certain degree, the TSC scheme inherently has a slightly higher reward than that of the TSC-free scheme. This can be seen in Figure 28 in the local enlarged image at the later stage of training. This graph shows the average reward at each step of training of the DRL agent. Figure 29 shows the maximum, minimum, and mean values of the total reward curve for each training episode of the research (TSC) and control (TSC-free) groups. The areas between the maximum and minimum are light green and light blue. We can clearly see the trend of the reward and the effect of the function convergence from this graph. It is worth mentioning that in Figure 28 and Figure 29, we normalized the initial positions of the TSC and TSC-free schemes to make them easier to display.

**Figure 30:**
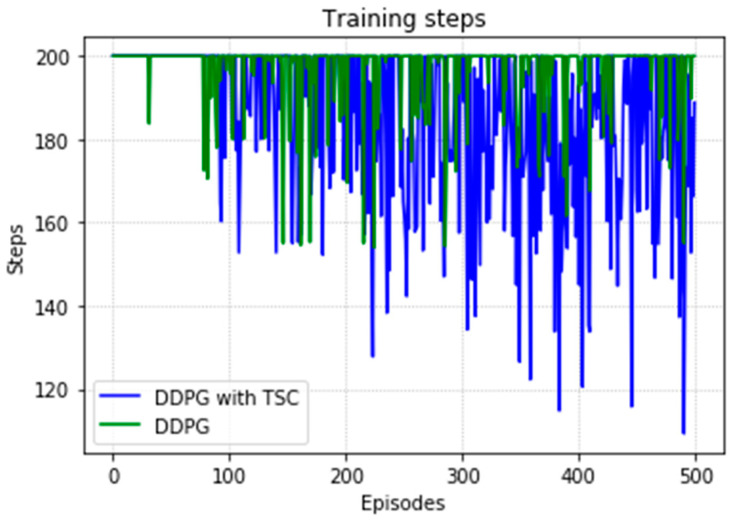
Training steps per training episode for the DDPG with and without TSC in the second experiment.

Figure 30 shows the number of training steps per training episode. The memory of the algorithm is 15,000, and thus the convergence effect is better after 75 generations because of 500 episodes and 200 steps. The DDPG with the TSC scheme outperforms the vanilla DDPG. The neural network achieves better results after being trained for 220 training episodes.

**Figure 31:**
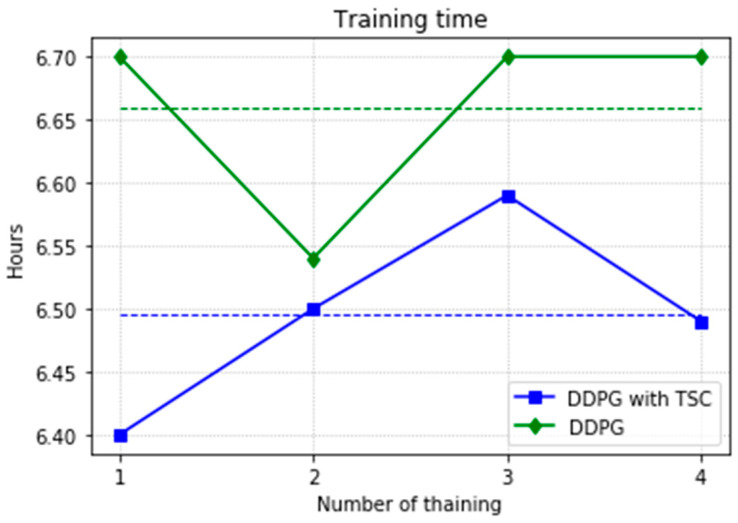
Training time for the DDPG with and without TSC in the second experiment.

Figure 31 shows a comparison of training time for the DDPG with and without TSC. The dotted line is the mean, which is the same as that in the first experiment. Due to limitations in computing power and time, only the second sample using the DDPG method converged to the global optimal solution when training for the maximum number of training episodes, and thus the time is relatively small; the time for the other three trials is 6.7 h. In contrast, the TSC scheme consumes relatively little time; this advantage will be more obvious if more training tests are performed.

At the 500th episode, the average reward increased by 10.75%, the total reward at each episode increased by 18.74%, the number of training steps decreased by 5.63%, and the average training time decreased by 2.48% compared with those for the vanilla DDPG.

**Figure 32:**
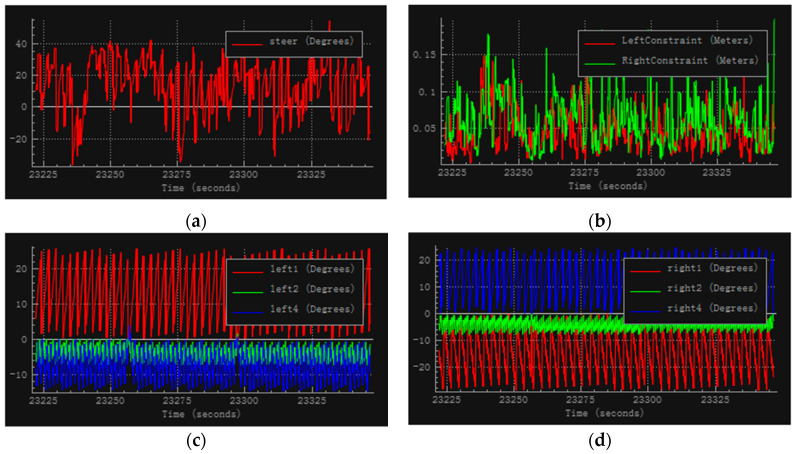
Simulation results in the second experiment. (**a**) Steering wheel angle, (**b**) distance from palm to steering wheel, (**c**) left arm joints, and (**d**) right arm joints.

The robot arm joint angles, steering wheel angle, and the distance from the palms to the steering wheel in the simulation obtained when the training of the neural network reached stability are shown in Figure 32. All simulation results are from data recorded by the V-REP simulation software during one of all trials. For each training cycle, the steering wheel reached about 30° and the restraint distance was within a few centimeters. This issue can be eliminated for an actual robot by using methods such as giving the robot a pair of gloves or increasing the diameter of the steering wheel.

The high sensitivity of vanilla DDPG to the hyperparameters affects the accuracy of the control methods and product development cycles in engineering problems. In the second experiment, we compared the performance of the TSC algorithm and the TSC-free algorithm (vanilla DDPG) on 17 groups of hyperparameters as shown in Table 5 (including the hyperparameter combination described in Table 4). The hyperparameters were composed of various combinations of learning rates of 0.001 and 0.005, discount factor of 0.6, 0.8 and 0.9, and batch sizes of 22~28. Owing to the limitations of computing power and time, each parameter combination was run only once.

Overfitting could have easily occurred according to knowledge of empirical risk minimization (ERM). The complexity of the neural network is related to the number of samples. The more joints the robot has, the more complex the network structure is, or the greater is the number of neurons needed. Taking Experiment 2 as an example, when the experience (replay buffer) constituted 15% of the total number of steps of evolution (the remaining experience was abandoned), the stability and speed of training reached a balance. The higher the reward discount is, the more we want the agent to pay more attention to the future. Usually, this is more challenging than “myopic;” and thus training is slightly more difficult. We also tested the program on two sets of learning rates. A hyperparameter that required a trade-off between efficiency and stability was also used. When 0.005 was used as the learning rate, the robot’s arm in early training quickly fitted a better level. However, the subsequent convergence was not good, and oscillations often occurred. For this reason, we used 0.001 as the learning rate in experiments described in the previous section.

**Figure 33:**
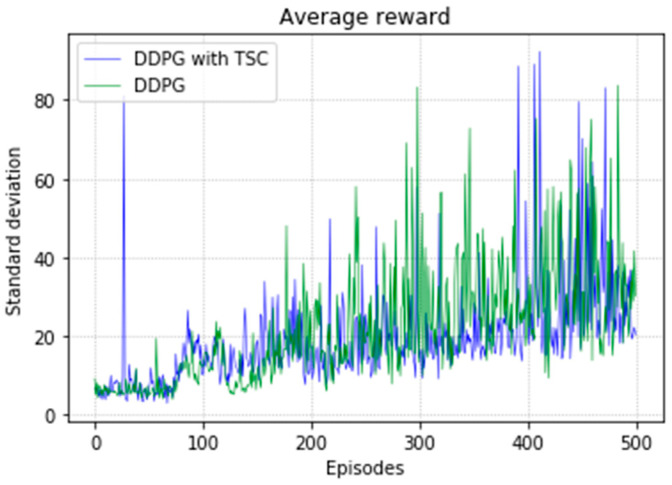
Standard deviation between the average reward of experiment 2 with different hyperparameters.

**Figure 34:**
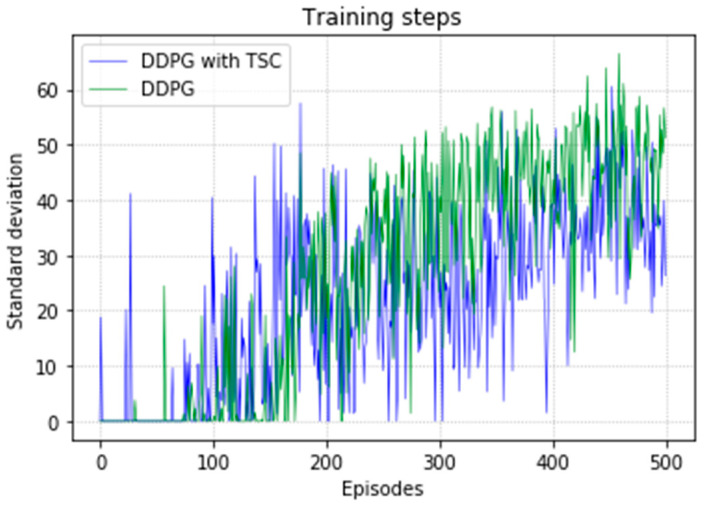
Standard deviation between the training steps of experiment 2 with different hyperparameters.

We used the same parameter combination to train the two methods separately and obtained comparative experimental results. Curves of the comparison between the experimental results of the methods on the 17 groups of hyperparameters are shown in Figure 33 and Figure 34. The blue line indicates the experimental group DDPG with the TSC method, and the green line is the vanilla DDPG. In the average reward shown in Figure 33, except for a large peak in the TSC method near the 30th episode, the standard deviation of the TSC method was significantly smaller than that of the TSC-free method for most of the time.

In Figure 34, before 160th episode of training, the standard deviation of the TSC method was large. Until the end of the training, the TSC method continued to yield better results than the TSC-free method.

## 6. Discussion and Future Work

In this work, we combined the DDPG algorithm with a sensor-based hardware system for human data acquisition. The control and trajectory planning of a planar 3-DOF manipulator and the 3D arms of the humanoid robot BHR based on the proposed algorithm were simulated. We have implemented strategies for the sampling of expert guidance in the form of hardware, which is novel in robot control.

It was difficult to obtain robust behavior when applying the vanilla DDPG to a real robot system. Although the scheme of the DDPG with TSC was somewhat unstable in rare cases, this instability is caused by the collected data and can be mostly ignored for an actual robot. In the remaining cases, the TSC scheme accelerated the convergence of deep neural network for the DRL algorithm.

When the DRL algorithm is applied to a robot, for a sufficient training time, the network can eventually converge to a good level. However, extremely high computing power is required and the training time is unacceptable for robotics applications, especially for general multi-tasking scenarios such as training robot arms to perform various actions, which requires the robot to learn in a relatively short period of time. The sensitivity of the DDPG to hyperparameters significantly affects the overall robot development cycle because researchers typically spend a lot of time adjusting the hyperparameters. With the TSC scheme, the DDPG becomes less sensitive to hyperparameters, even with the addition of new adjustable reward function coefficients; as long as the value of the reward function is set within a reasonable range, the algorithm can converge well.

Our research explored a new way of combining DRL with sensors and mechanical systems. A large part of the work in this study was the development of the data acquisition system, which involved sensors, analog and digital electronics, mechanics, and mechanical component design. After all the rotating joints are made and adjusted, they need to be improved on the shelf to adapt to the changing laws of human joints as much as possible. Although this process seems time-consuming, once debugging is complete, it speeds up the planning of manipulator movements for various tasks. Although the development of the data acquisition system required effort, it will shorten the planning cycle for future robot motion development. The acquisition system is a universal humanoid, making it applicable to several kinds of humanoid robot.

The TSC scheme introduced in this paper needs some improvement. The mechanical structure requires the most modification. More accurate tracking of the changes in human joint rotation by the potentiometers would result in a better effect of the DRL algorithm training. Another area for improvement is the service life of the sensors, which use graphite. The simulation environment can also be improved. The BHR humanoid robot we set up in the simulation can be modeled more accurately to reduce the gap between simulation and reality. Computing power can be increased to reduce calculation time. In future work, in addition to solving the above problems, we will apply the actions planned in the simulation to an actual robot to test the reliability of the TSC scheme in an actual environment. We will also conduct research on DRL for walking stability control of humanoid robots.

## Figures and Tables

**Table 1:** Joint rotation angles input to DDPG by TSC.

***T_i_***	${\hat{\theta }}_{O}$	${\hat{\theta }}_{A}$	${\hat{\theta }}_{B}$
*T_1_*	−50°	84°	51°
*T_2_*	−45.7°	89.9°	40.8°
⋮	⋮	⋮	⋮
*T_50_*	130°	91°	79°

**Table 2:** Hyperparameters of the model.

DDPG Setup Hyper-Parameters	
Actor/Critic learning rate	1 × 10^−3^
Reward discount factor γ	0.9
Soft replacement τ	0.01
Batch size	32
Running episodes	300
Number of track points K	50
Training steps per update	200
Memory capacity	80,000
Updates	*episodes* × *points* × *steps*

**Table 3:** Specifications of robotic arm.

Items	Specifications				
Range constraints of the joints	Left arm	Shoulder	Joint 1	Pitch	−20°~20°
			Joint 2	Roll	−5°~10°
		Elbow	Joint 3	Pitch	−10°~60°
	Right arm	Shoulder	Joint 1	Pitch	−20°~20°
			Joint 2	Roll	−5°~10°
		Elbow	Joint 3	Pitch	−10°~60°
	Steering wheel			−90°~90°	

**Table 4:** Hyperparameters for robot driving.

Algorithm Setup Hyper-Parameters	
Actor/Critic learning rate	0.001
Reward discount factor δ	0.9
Soft replacement τ	0.001
Batch size	32
Running episodes	500
Steps per episode	200
Memory capacity	15,000
Updates	*episodes* × *steps*
Angle factor α	0.5
Distance factor β	0.5
TSC factor γ	20
Control cycle (s)	0.125

**Table 5:** Hyperparameters experiment table.

Hyper-Parameters	Actor/Critic Learning Rate	Reward Discount Factor	Batch Size
1	0.001	0.6	32
2	0.001	0.8	16
3	0.001	0.8	32
4	0.001	0.8	64
5	0.001	0.8	128
6	0.001	0.8	256
7	0.001	0.9	16
8	0.001	0.9	32
9	0.001	0.9	64
10	0.001	0.9	128
11	0.001	0.9	256
12	0.005	0.9	16
13	0.005	0.9	32
14	0.005	0.9	64
15	0.005	0.8	64
16	0.005	0.9	128
17	0.005	0.9	256
